# Models of integrated care for multi-morbidity assessed in systematic reviews: a scoping review

**DOI:** 10.1186/s12913-023-09894-7

**Published:** 2023-08-23

**Authors:** Anke Rohwer, Ingrid Toews, Jeannine Uwimana-Nicol, John L.Z. Nyirenda, Jean Berchmans Niyibizi, Ann R. Akiteng, Joerg J. Meerpohl, Charlotte M. Bavuma, Tamara Kredo, Taryn Young

**Affiliations:** 1 Centre for Evidence-based Health Care, Division Epidemiology and Biostatistics, Department of Global Health, Faculty of Medicine and Health Sciences, Stellenbosch University, Cape Town, South Africa; 2 Institute for Evidence in Medicine (for Cochrane Germany Foundation), Faculty of Medicine, Medical Center - University of Freiburg, University of Freiburg, Freiburg, Germany; 3 College of Medicine and Health Sciences, University of Rwanda, Kigali, Rwanda; 4 College of Health Sciences, Makerere University, Kampala, Uganda; 5 Cochrane Germany, Cochrane Germany Foundation, Freiburg, Germany; 6 Kigali University Teaching Hospital, Kigali, Rwanda; 7 South African Medical Research Council, Cochrane South Africa, Cape Town, South Africa; 8 Division of Clinical Pharmacology, Department of Medicine, Faculty of Medicine and Health Sciences, Stellenbosch University, Cape Town, South Africa

**Keywords:** Integrated care, Multi-morbidity, Chronic diseases, Non-communicable diseases, Low- and middle-income countries, Systematic review, Collaborative care

## Abstract

**Background:**

The prevalence of multi-morbidity is increasing globally. Integrated models of care present a potential intervention to improve patient and health system outcomes. However, the intervention components and concepts within different models of care vary widely and their effectiveness remains unclear. We aimed to describe and map the definitions, characteristics, components, and reported effects of integrated models of care in systematic reviews (SRs).

**Methods:**

We conducted a scoping review of SRs according to pre-specified methods (PROSPERO 2019 CRD42019119265). Eligible SRs assessed integrated models of care at primary health care level for adults and children with multi-morbidity. We searched in PubMed (MEDLINE), Embase, Cochrane Database of Systematic Reviews, Epistemonikos, and Health Systems Evidence up to 3 May 2022. Two authors independently assessed eligibility of SRs and extracted data. We identified and described common components of integrated care across SRs. We extracted findings of the SRs as presented in the conclusions and reported on these verbatim.

**Results:**

We included 22 SRs, examining data from randomised controlled trials and observational studies conducted across the world. Definitions and descriptions of models of integrated care varied considerably. However, across SRs, we identified and described six common components of integrated care: (1) chronic conditions addressed, (2) where services were provided, (3) the type of services provided, (4) healthcare professionals involved in care, (5) coordination and organisation of care and (6) patient involvement in care. We observed differences in the components of integrated care according to the income setting of the included studies. Some SRs reported that integrated care was beneficial for health and process outcomes, while others found no difference in effect when comparing integrated care to other models of care.

**Conclusions:**

Integrated models of care were heterogeneous within and across SRs. Information that allows the identification of effective components of integrated care was lacking. Detailed, standardised and transparent reporting of the intervention components and their effectiveness on health and process outcomes is needed.

**Supplementary Information:**

The online version contains supplementary material available at 10.1186/s12913-023-09894-7.

## Background

Globally, the prevalence and burden of multi-morbidity, the existence of two or more chronic health conditions in one individual [1], is increasing [2]. Multi-morbidity refers to people with multiple non-communicable diseases (NCDs) such as cardiovascular diseases, cancers, chronic respiratory diseases and diabetes, [3] mental health conditions and communicable diseases. Although NCDs are a global problem, low-and middle-income countries (LMICs) are hit the hardest, where more than 75% of global deaths due to NCDs occur [3]. In LMICs, the burden of multi-morbidity may be exacerbated by emerging infections such as COVID-19 and the double burden of NCDs and chronic communicable diseases, such as Human immunodeficiency virus (HIV) infection and tuberculosis (TB). The prevalence of multi-morbidity in LMICs ranges from 13 to 87% and is expected to increase over time if there are no drastic measures for preventing and controlling chronic diseases [4, 5]. Life expectancy of people living in LMICs has improved over the past two decades and a considerable number of people are reaching middle and older ages when NCDs such as cardiovascular diseases, type 2 diabetes mellitus, and cancers, among others are increasing. Furthermore, people living in LMICs are also experiencing a change in lifestyle and environmental exposures which contribute to NCDs.

Management of and care for patients with multi-morbidity is often fragmented, as specialised care is needed to address each individual condition. This often involves both primary and multiple secondary care specialists who may not be communicating and coordinating care effectively with resultant impact on health outcomes and impact on households [6]. To respond to the growing burden caused by multi-morbidity and to meet international health targets, models of care that enhance the continuum of care, adherence to care, reduce number of health visits and multidisciplinary management should be prioritised [7].

Integrated approaches or models of care are described as providing patients with holistic options centred on health needs of people and communities and thereby enhancing community self-reliance [8–12]. Various approaches, models of care and frameworks, aiming to improve health outcomes and strengthen health systems, have been described [13]. As an example, the WHO framework on ‘integrated, people-centred health services’ [14] and the ‘conceptual and analytical framework on integrated care for health programs’ developed by Atun et al. 2010 [8] provide an analytical approach that helps researchers to apply the framework when conducting evaluative and formative studies on ‘integration’ in order to generate useful evidence to inform policy and practice in different health setting [8, 12].

Integrated care has been widely promoted to help provide services for people with multi-morbidity assuming that they achieve more appropriate, better-quality as well as less resource-intensive and therefore more cost-effective care. However, many evidence gaps related to the prevention and management of multi-morbidity remain [15]. Many different definitions and models of integrated care exist, and it is often difficult to unpack the components and mechanisms of action of these complex interventions [16]. Furthermore, it is still unclear which components and characteristics of integrated care render it more effective than other models of care.

Initially, we set out to conduct an overview of systematic reviews on the effects of integrated models of care. However, during the process of identifying studies for inclusion, the author team realised that the complexity and heterogenous nature of integrated models of care would make it difficult to compare effects across SRs. We decided that it was important to understand the various ways integrated care has been defined and reported in SRs as a first step to then inform further work on effects. We therefore conducted a scoping review, which is better suited for this objective [17], and aimed to describe and map the definitions, characteristics, components and effects of integrated models of care as reported in SRs.

## Methods

We developed a protocol for an overview of SRs (PROSPERO: CRD42019119265), which we adapted for this scoping review. We followed the pre-specified methods linked to eligibility criteria, identification of reviews including the search strategy, and selection of reviews, but adapted the methods linked to data extraction and analysis to fit with the objectives of this scoping review. We used the PRISMA Extension for Scoping Reviews to guide reporting [18].

### Eligibility criteria

We included SRs on integrated models of care at either primary health care (PHC) level only or both PHC and specialized health settings for adults and children with multi-morbidity. We focused on PHC, as effective management of chronic conditions requires a shift from curative to preventative, and from inpatient to outpatient care [14]. We were therefore not interested in treatment of acute complications in a hospital setting, but in long-term management of chronic conditions. Multi-morbidity was defined as having two or more chronic conditions. We considered the following NCDs and communicable diseases to be chronic conditions: Diabetes mellitus, cardiovascular diseases, cancers, chronic respiratory diseases, mental diseases (e.g. depression), musculoskeletal disorders (e.g. Arthritis), chronic kidney disease, HIV, and TB. The key characteristics of SRs were defined as having a clearly stated set of objectives with an explicit and reproducible methodology; a systematic search that attempts to identify all studies that would meet the eligibility criteria; an assessment of the validity of the findings of the included studies (e.g., assessment of risk of bias and confidence in cumulative estimates); and systematic presentation, and synthesis of the characteristics and findings of the included studies [19]. We therefore considered a review to be a SR if it included (1) pre-specified objectives and eligibility criteria of studies; (2) a search of at least two electronic databases to identify studies; and (3) assessment of risk of bias of included studies.

**Fig. 1:**
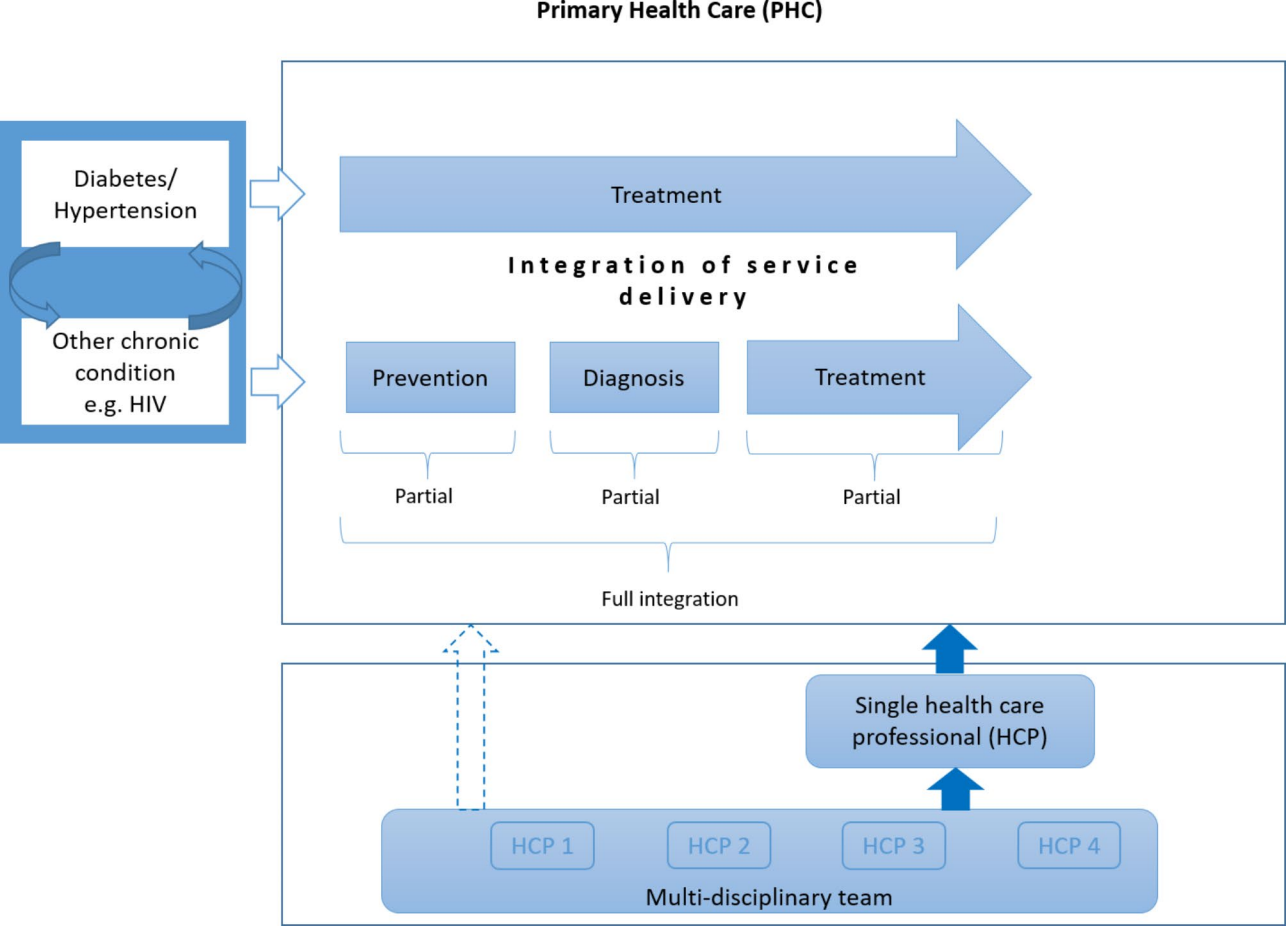
Logic model of integrated care [20]

We included interventions that comprised fully integrated care or partially integrated care. Full integration of care referred to models where patients (primarily treated for one condition) receive the full package of care (prevention, diagnosis and treatment) for another condition at the same point of care by one or more health care professionals. Partial integration of care was defined as models where patients treated for one condition receive part of the package of care (either prevention, diagnosis, or treatment) for another condition (Fig. 1). As a minimum, patients had to receive preventative measures such as health education or be screened (and referred if they received a positive test result) for another specific condition for the intervention to be classified as integrated care. Studies that did not adequately define their intervention were included if it was clear that the intervention aimed to integrate care for two or more chronic conditions.

### Information sources and search

We conducted a comprehensive search of PubMed (MEDLINE), Embase, Cochrane Database of Systematic Reviews, Epistemonikos, and Health Systems Evidence up to 3 May 2022. Keywords included ‘Chronic diseases’, ‘Integrated health care’, ‘Systematic review’ and their synonyms. We did not apply restrictions based on language or date of publication. The full search strategies for all databases are provided in Additional file 1.

### Selection of systematic reviews

A pair of authors (AR, IT, JLZN, JUN, JBN) independently screened titles and abstracts in duplicate, using Covidence software and obtained full texts of potentially relevant articles. A pair of authors (AR, IT, JLZN, JUN, JBN) independently screened full texts according to the pre-specified eligibility criteria and provided reasons for excluding studies. Discrepancies were resolved through discussion with another member of the author team.

### Data extraction and analysis

A pair of authors (AR, JUN, IT, JBN, AA, JLZN) independently extracted data using a pre-piloted data extraction form set up in Covidence. We extracted descriptive characteristics of included SRs, comprising objectives of the SR; characteristics of included participants, interventions and comparisons; how integrated care was defined; key features of integrated care; the outcomes addressed; eligible study designs and actual number and type of studies included; country and setting where included studies were conducted; databases searched and the date of the last search. Discrepancies were resolved through discussions with the author team.

We summarised descriptive characteristics of included SRs narratively. We mapped available evidence in table format, focussing on the characteristics of various models of integrated care included in the SRs. We extracted information on main components and sub-components of models of integrated care across SRs and tabulated these to compare models. We identified common components of integrated models of care through iterative discussions within the author team. We extracted findings of the SRs as presented in the conclusions and reported on these verbatim.

## Results

### Results of search and description of included studies

**Fig. 2:**
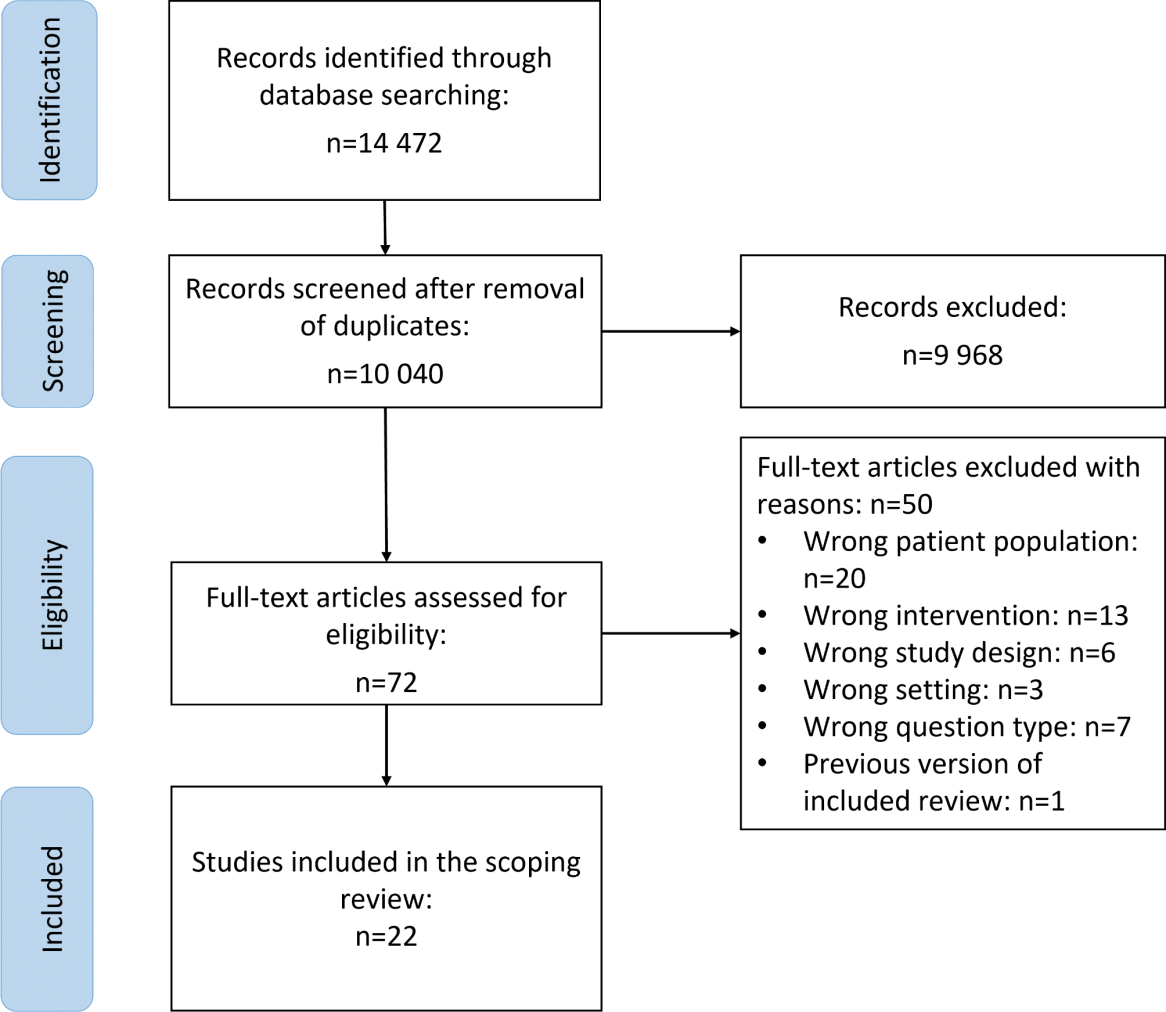
PRISMA Flow diagram of included studies

After removal of duplicates, we screened titles and abstracts of 10,040 records and assessed eligibility of 72 full texts. We included 22 SRs and excluded 50 reviews with reasons (Additional file 2). The PRISMA flow-diagram is depicted in Fig. 2.

**Fig. 3:**
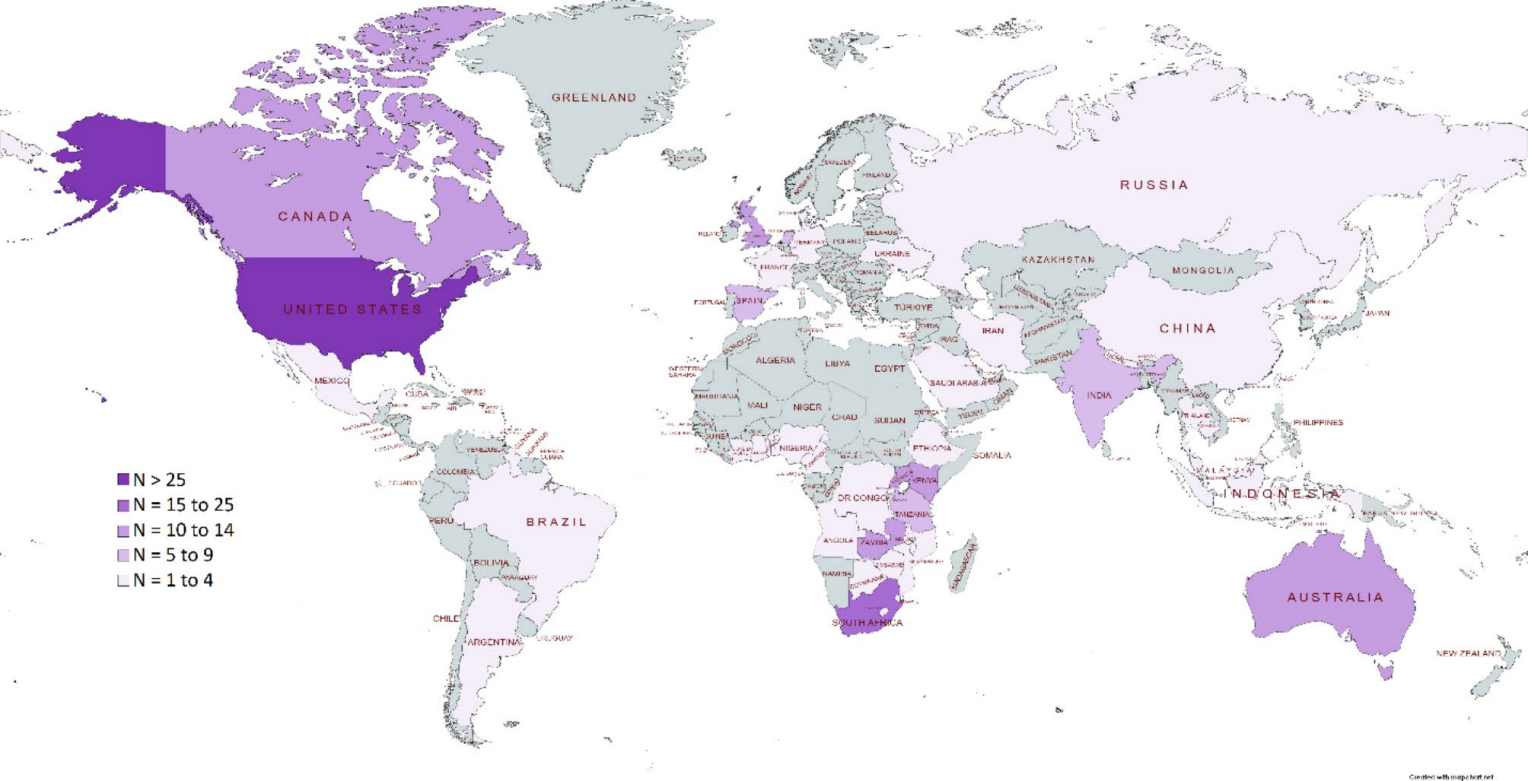
Heatmap of primary studies included in the SRs

The included SRs [21–42] were published between 2011 and 2022, with the dates of the last search ranging from September 2010 to October 2021 (Table 1). Primary studies included in the SRs were exclusively conducted in high income countries (HICs) in twelve SRs [21, 25–28, 30–33, 37, 39, 42] and exclusively in LMICs in six SRs [23, 29, 34, 36, 40, 41]. Four SRs included studies from LMICs and HICs [22, 24, 35, 38] (Fig. 3). Overall, the included SRs examined data from randomised controlled trials (RCTs), controlled before-after studies, non-randomised intervention studies, interrupted time series, cohort studies, case series, cross-sectional studies, retrospective record reviews, mixed-methods studies, quasi-experimental studies, and qualitative studies. Data was also derived from program descriptions in one SR. Ten SRs included RCTs only [21, 26, 28, 30–33, 35, 37, 42].

**Table 1:** Summary characteristics of included systematic reviews

Study ID	Date of last search	Type and number of studies included	Country of included studies	Participants/ Conditions included	Intervention	Health outcomes addressed
Atlantis 2014 [21]	August 2013	Randomised control trials (RCTs) (n = 7)	USA (n = 6) Australia (n = 1)	Adults with comorbid depression and diabetes	Collaborative care	• Depression • HbA1C
Bulstra 2021 [34]	September2021	RCTs (n = 4) Interrupted time series (n = 2) Pre-post studies (n = 12) Retrospective cohort studies (n = 9) Prospective cohort studies (n = 7) Cross-sectional studies (n = 5) Prospective longitudinal controlled intervention study (n = 1) Modelling study (n = 1)	South Africa (n = 11) Ghana (n = 1) Uganda (n = 3) USA (n = 5) Malawi (n = 3) Ethiopia (n = 1) Zambia (n = 5) sub-Saharan Africa (9 countries not specified) (n = 1) Tanzania (n = 2) Cameroon (n = 1) Ukraine (n = 1) Mozambique (n = 1) Rwanda (n = 1) Kenya (n = 1) Eswatini (n = 1) Democratic Republic of the Congo (n = 1) Denmark (n = 1) China (n = 1)	Adults and adolescents with HIV and TB, or with HIV and seeking other health services including maternal and child health family planning, primary health care and sexual and reproductive health or sexually transmitted infection services	Bi-directional integration of HIV services into non-HIV programmes and non-HIV services into HIV programmes	• Uptake of HIV services • HIV testing yield • ART initiation • Time until ART Initiation • Retention in care • ART adherence • Viral suppression • HIV – free survival among infants • HIV infections averted • AIDS related mortality • Uptake of other health services • Treatment success for other diseases/ conditions • Non-AIDS related mortality • HIV only costs • Non-HIV costs • Costs of integrated services versus HIV only costs • Cost effectiveness
Chuah 2017 [22]	October 2015	RCTs (n = 7) Non-randomized intervention studies (n = 5) Cohort studies (n = 5) Case-series studies (n = 3) Cross-sectional (n = 3) Retrospective record reviews (n = 3) Mixed-method studies (n = 3) Programme or model descriptions (n = 14) Qualitative methods (n = 2)	USA (n = 32) UK (n = 3) Canada (n = 1) Australia (n = 1) France (n = 1) South Africa (n = 2) Uganda (n = 3) Zimbabwe (n = 1) Tanzania (n = 1)	Adults with comorbid HIV and at least one mental disorder	Integration of HIV care into mental health services, or integration of mental health services in HIV care	• Depression • Mental health problems • Alcohol and substance use • Social functioning • HIV symptoms • Viral suppression • CD4 count • HIV stigma • Risk behaviour • HIV knowledge • HIV adherence • Health-related quality of life
Dudley 2011 [23]	September 2010	RCTs (n = 5) Controlled before-after studies (CBAs) (n = 4)	India (n = 2) Tanzania (n = 1) Zambia (n = 1) Nepal (n = 1) Togo (n = 1) South Africa (n = 2) Zimbabwe (n = 1)	Patients using primary health care services	Integration of 1) family planning and immunisation (n = 1); maternal and child health services (n = 2); HIV counselling and testing (n = 1) 2) Integration of nutrition and infectious disease control (n = 1) 3) Integration of STI, HIV and TB services (n = 1)	• Health-care delivery • User views • Knowledge and behaviours of service users, • Health status • Users’ perceptions of the service
Haldane 2018 [24]	October 2015	Cohort study (n = 1) Retrospective record review (n = 2) Program description (n = 12) Cross-sectional study (n = 1) Qualitative study (n = 1)	USA (n = 4) UK (n = 1) Ethiopia (n = 1) Uganda (n = 3) Cambodia (n = 1) South Africa (n = 1) Kenya (n = 4) Nigeria (n = 1) Lesotho (n = 1)	Patients with HIV/AIDS and diabetes, hypertension or cardiovascular disease risk factors	Integration of HIV/AIDS and chronic disease services	• Blood pressure • HbA1C • Cholesterol levels • CD4 count
Hopman 2016 [25]	March 2014	Included a total of 18 studies, of which 4 are relevant to this scoping review: RCTs (n = 3) Cohort study (n = 1)	USA (n = 3) Canada (n = 1)	Patients with multiple chronic diseases	Comprehensive care	• Patient satisfaction • Health related quality of life • Depressive symptoms • Functional status • Mortality
Huang 2013 [26]	March 2013	RCTs (n = 8)	USA (n = 8)	Patients with comorbid depression and diabetes	Collaborative care	• Depression treatment response • Depression remission • HbA1c control • Adherence
John 2020 [35]	March 2020	RCTs (n = 28)	USA (n = 14) Netherlands (n = 5) South Africa (n = 1) Puerto Rico (n = 1) UK (n = 4) Germany (n = 1) Spain (n = 2)	Primary care patients with diagnosis of one or more chronic conditions	Integrated or multi-disciplinary care	• Blood pressure • Glycated haemoglobin (HbA1c) • Low density lipoprotein cholesterol (LDL-C) • High density lipoprotein cholesterol (HDL-C) • Serum total cholesterol • Depression • Health-related quality of life • Self-management • Hospital admissions • Emergency department visits • Medications use • Incremental cost-effectiveness ratio (ICER)
Kadia 2021 [36]	July 2019	Prospective cohort studies (n = 6) Retrospective cohort studies (n = 12) Prospective and retrospective cohort study (n = 1) Cross-sectional studies (n = 3) Case study (n = 1)	Uganda (n = 2) Benin (n = 1) Malawi (n = 5) Rwanda (n = 2) Zambia (n = 1) South Africa (n = 2) Kenya (n = 3) Cameroon (n = 1) Ghana (n = 1) Democratic Republic of the Congo (n = 2) Eswatini (n = 1) Zimbabwe (n = 1) Ethiopia (n = 1)	TB/HIV co-infected adult patients	Collaborative care	• ART uptake • barriers to ART uptake • enablers of ART uptake
Kappelin 2021 [37]	December 2019	RCTs (n = 12)	Spain (n = 1) USA (n = 6) England (n = 1) Canada (n = 1) Netherlands (n = 1) Puerto Rico (n = 1) Australia (n = 1)	Adults with mental health issues and one physical diseases	Collaborative Care	• Improvement in depressive symptoms • Improvement in anxiety symptoms
Kastner 2018 [27]	December 2017	RCTs (n = 15) Cluster RCTs (n = 6) Mixed methods studies (n = 3) Uncontrolled studies (n = 1)	USA (n = 11) Australia (n = 7) Canada (n = 1) Spain (n = 1) Germany (n = 1) Russia (n = 1) The Netherlands (n = 1) Other European countries (n = 2)	Adults with multi-morbidity	Multi-morbidity interventions	• Depression • HbA1C • Systolic blood pressure • Mortality • Quality of life • Antidepressant use • Physical activity
Lee 2021 [38]	March 2018	RCT (n = 15) Prospective cohort studies (n = 7) Retrospective cohort (n = 1) prospective pre-post studies (n = 2) Retrospective pre-post (n = 14)	Mexico (n = 2) Brazil (n = 4) USA (n = 18) Canada (n = 4) Taiwan (n = 1) Israel (n = 1) Malaysia (n = 1) Saudi Arabia (n = 2) Hong Kong (n = 2) The Netherlands (n = 1) Iran (n = 1) Australia (n = 1) American Samoa (n = 1)	Adults with Diabetes and Hypertension	Interprofessional collaborative practice	• (HbA1c) • Systolic blood pressure (SBP) • Diastolic blood pressure (DBP) levels
Li 2017 [28]	January 2015	RCTs (n = 25); of which 7 assessed collaborative care interventions (relevant to this scoping review)	USA (n = 4) Scotland (n = 3)	Adult cancer patients with major depression or other non-bipolar depressive disorders	Collaborative care	• Depression
Martens 2021 [39]	January 2020	RCT (n = 8) Quasi-experimental study (n = 1)	Denmark (n = 1) USA (n = 7) Spain (n = 1)	Persons with severe mental illness and at least one chronic condition	Implementation of organizational models of care	• Body weight • Body Mass Index • HbA1C • Blood glucose • Cardiovascular risk • Low density lipids • Total cholesterol • Systolic blood pressure • Diastolic blood pressure • Quality of life • Number of screening visits
Nyirenda 2022 [40]	October 2021	RCT (n = 1) Prospective cohort study (n = 1) Cross-sectional studies (n = 4) Prospective observational (n = 3) Retrospective cohort (n = 1)	Mexico (n = 1) China (n = 3) India (n = 2) Indonesia (n = 1) Zimbabwe (n = 1) Angola (n = 1) Uganda (n = 1)	Patients with tuberculosis and/or diabetes	Integrated care for TB and Diabetes	• Screening coverage • Treatment loss to follow-up • Blood glucose control • Cure rate
Rohwer 2021 [41]	December 2019	Cluster RCTs (n = 3) Interrupted time series study (n = 2)	South Africa (n = 3) Uganda/Kenya (n = 1) India (n = 1)	Adults and children with diabetes and hypertension	Full or partial integration of services at PHC and community level	• All-cause mortality • Blood pressure control • NCD control • HIV control • Access to care • Depression • Quality of life • HbA1C • Systolic blood pressure • Total cholesterol • Adherence • Retention in care • Quality of care
Sigfrid 2017 [29]	December 2015	Cohort studies (n = 4) Cross-sectional studies (n = 15) Retrospective record reviews (n = 3) Before-after study (n = 1)	Kenya (n = 3) Uganda (n = 1) Mozambique (n = 1) Zambia (n = 4) Ivory Coast (n = 2) Tanzania (n = 3) Guyana (n = 3) Thailand (n = 1) UK (n = 1) Nigeria (n = 1) Argentina (n = 1) Botswana (n = 1) Ethiopia (n = 1)	Women with HIV and cervical cancer	Integrated care	• Cervical cancer screening • Referral • Cryotherapy • Colposcopy • Pathology results • Cancer diagnosis • CD4 counts • Proportion on ART • Sexually transmitted infections • HIV screening
Smith 2021a [30]	September 2015	RCTs (n = 18)	USA (n = 16) UK (n = 1) Canada (n = 1)	Adults with multi-morbidity	Interventions designed to improve multi-morbidity	• Physical health • Mental health • Psychosocial outcomes • Health service use • Patient related behaviours • Medication adherence
Smith 2021b [42]	September 2019	RCTs (n = 9)	Germany (n = 1) UK (n = 2) USA (n = 4) Canada (n = 1) Spain (n = 1)	Adults with multimorbidity	Coordinated care	• Health service use • Self-management (Health Education Impact Questionnaire) • Emergency admissions • Self-management • Health-related quality of life • Well-being (W-BQ12) • Number medications • Health services use • Self-rated health • Dietary behaviour • Physical activity • Activity participation • Quality of medication therapy • Pharmaceutical care issues • Medication Appropriateness Index
Tully 2015 [31]	April 2014	RCTs (n = 6)	USA (n = 5) Australia (n = 1)	Adults with comorbid depression and coronary heart disease	Collaborative care	• Major adverse cardiac events • Depression symptoms • Depression remission • Anxiety
Van Eck van der Sluijs 2018 [32]	August 2017	RCTs (n = 20)	USA (n = 18) UK (n = 1) The Netherlands (n = 1)	Adults with a chronic medical condition and a depressive and/or anxiety disorder	Collaborative care	• Symptom-load related to chronic medical condition • Incidence of MACE, angina pectoris, post infarct • Arthritis-related pain • Arthritis-related physical functioning • Cancer-related pain • Cancer-related physical functioning • Dyspnea related disability • HbA1C • Epilepsy-related seizures • HIV symptom severity • Blood pressure • Physical functioning • Depressive symptoms
Watson 2013 [33]	June 2012	RCTs (n = 12)	USA (n = 11) UK (n = 1)	Patients with depression and one or more chronic conditions	Practice-based interventions that include coordinated care, integrated care and collaborative care	• Depression • Symptom improvements • Depression-free days • Remission • Recurrence • Treatment adherence • Treatment satisfaction • Use of antidepressants • Mental health-related Quality of life • Mental Health care utilisation

The included SRs (Table 1) looked at a wide array of medical conditions that were addressed with collaborative care or integrated care approaches. These included HIV, TB, NCDs, diabetes, hypertension, cardiovascular disease, cancer, depression and other mental health conditions. Some reviews reported that they considered multiple conditions or patients with multi-morbidity.

Included SRs provided various definitions for integrated care, ranging from very simple to very comprehensive definitions (Additional file 3). The different models for delivering care and offering health services were described as integrated care [22–24, 27, 29, 30, 34, 36, 40, 41] or collaborative care [21, 26, 28, 31, 32, 39], with a few SRs reporting the models of care as comprehensive care [25, 33], a multi-professional approach to patient care [37], interprofessional collaboration practice [38] and patient-centered medical home [35].

### Characteristics and components of models of integrated care

Models of care evaluated in primary studies were heterogenous within and across SRs. We identified six common components across included SRs, related to (1) chronic conditions addressed, (2) where services were provided (3) types of services provided (4) healthcare professionals involved in care (5) coordination and organisation of care and (6) patient involvement in care. Within each of these components, we identified various sub-components reported across included SRs (Table 2). Additional file 4 provides additional details for each included review.

**Table 2:** Components of integrated care as reported in systematic reviews

Study ID	Components of integrated care related to:					
	Conditions	Where services were provided	Types of services provided	Health professionals involved in care	Coordination and organisation of care	Involvement of patients in care
**Systematic reviews with included studies from low- and middle-income countries**						
Bulstra 2021 [34]	HIV and TB, diabetes, hypertension, cancer, NCDs, mental health	Single facility: • One-stop-shop • Co-location Multiple facilities	Health education and counselling Screening Diagnosis Linkage to care Treatment	Not reported	Not reported	Not reported
Dudley 2011 [23]	HIV and TB	Single facility	Health education and counselling Screening Diagnosis Treatment	Other HCP	Not reported	Not reported
Kadia 2021 [36]	HIV and TB	Single facility	Treatment for HIV and TB	Not reported	Not reported	Not reported
Nyirenda 2022 [40]	TB and Diabetes	Single facility	Screening	Not reported	Not reported	Not reported
Rohwer 2021 [41]	Diabetes and/or hypertension and HIV, depression	Single facility: • Co-location • One-stop-shop Community	Health education and counselling Screening Diagnosis Linkage to care Treatment	Nurse General physician Other HCPs	Coordination of care Clinical management support Staff support Facility reorganisation	Not reported
Sigfrid 2017 [29]	HIV and cervical cancer	Single facility: • One-stop-shop • Co-location Multiple facilities	Health education and counselling Screening Treatment Referral	Nurse Specialists	Coordination of care	Not reported
**Systematic reviews with included studies from low-, middle-, and high-income countries**						
Chuah 2017 [22]	HIV and mental health	Single facility	Not reported	Not reported	Coordination of care Interprofessional communication	Not reported
		Multiple facilities	Referral	Not reported	Coordination of care Interprofessional communication	Not reported
		Not reported	Referral	Nurse Other HCP	Case management	Patient engagement
Haldane 2018 [24]	HIV and CVD, hypertension or diabetes	Single facility	Treatment Screening Referral	MDT	Interprofessional communication	Patient engagement Self-management
John 2020 [35]	Multiple conditions	Not reported	Not reported	MDT	Coordination of care Interprofessional communication	Patient engagement Self-management
Lee 2021 [38]	Diabetes and hypertension	Single facility: • Co-location	Health education and counselling	MDT	Coordination of care Interprofessional communication Case management Clinical management support Structured treatment plans	Patient engagement Self-management
**Systematic reviews with included studies from high-income countries**						
Atlantis 2014 [21]	Diabetes and depression	Not reported	Health Education and counselling Treatment Referral	Nurse Other HCP	Case management Structured treatment plan	Patient engagement Self-management
Hopman 2016 [25]	Multiple chronic conditions	Single facility Home	Not reported	Nurse General physician MDT	Coordination of care Interprofessional communication Case management Clinical management support	Self-management
Huang 2013 [26]	Depression and diabetes	Single facility	Treatment	Nurse General physician MDT	Coordination of care Interprofessional communication Structured management plan	Not reported
Kappelin 2021 [37]	Multiple conditions	Not reported	Treatment	Nurse General physician Other HCP	Coordination of care Interprofessional communication Structured management plans	Self-management
Kastner 2018 [27]	Diabetes, CVD, depression, NCDs	Not reported	Health education and counselling Treatment	MDT	Case management Clinical management support Structured treatment plans	Self-management
Li 2017 [28]	Cancer and depression	Home	Treatment	Not reported	Coordination of care	Self-management
Martens 2021 [39]	Mental health and multiple conditions	Not reported	Health education and counselling Screening Treatment	Nurse Specialist Other HCP MDT Peers	Coordination of care Interprofessional communication Case management Structured treatment plans Staff support	Patient engagement Self-management Peer support
Smith 2021a [30]	Multiple chronic conditions	Single facility Multiple facilities Home	Health education and counselling Diagnosis Treatment	Nurse General physician Specialist Other HCP MDT Peers	Coordination of care Interprofessional communication Case management Structured management plans	Patient engagement Self-management
Smith 2021b [42]	Multiple chronic conditions	Not reported	Not reported	Nurse General physician Other HCP MDT	Coordination of care Case management Structured treatment plans Staff support	Patient engagement Self-management Communication with HCP
Tully 2015 [31]	Coronary heart disease and Depression	Not reported	Health education and counselling Treatment Referral	Nurse General physician Specialist Other HCP MDT	Coordination of care Structured treatment plans	Patient engagement Communication with HCP
Van Eck van der Sluijs 2018 [32]	Chronic diseases and depression or dysthymia	Single facility	Diagnosis Treatment	General physician Specialist Other HCP	Case management	Not reported
Watson 2013 [33]	Chronic diseases and depression	Not reported	Health education and counselling Treatment Referral	Nurse Specialist Other HCP MDT	Structured treatment plans	Patient engagement Self-management

Specific c*hronic conditions addressed* in systematic reviews included HIV, TB, diabetes mellitus, hypertension, cardiovascular disease, cancer and mental health (mostly depression). Some reviews only referred to NCDs or multiple conditions collectively.

*(Where) Services were provided* at a single facility, multiple facilities, and at home or in the community. Where services were provided at a single facility, these were described as ‘within clinic’ or as ‘co-location’. Services offered within the same clinic were referred to as a ‘one-stop-shop’, where services related to all conditions were provided at the same time, by the same healthcare professional. In contrast, ‘co-location’ referred to services provided in different clinics, by different healthcare professionals, but at the same facility.

*Types of services provided* included health education and counselling, screening, diagnosis, linkage to care, treatment and management of conditions, referral, appointment reminders and telephonic follow-up. *Healthcare professionals involved in care* included primary care nurses, general physicians, specialists, other healthcare professionals such as physiotherapists, psychologists or pharmacists, a multi-disciplinary team, peers, or a combination of these.

We identified various sub-components for *coordination and organisation of care*. The sub-component coordination of care described internal and external referral systems, scheduled follow-ups, and continuity of care. Interprofessional communication comprised regular team meetings, case discussions, enhanced communication, joint consultations, and shared medical records. Case management referred to appointment of case managers. Clinical management support comprised clinical practice guidelines, algorithms and decision-aids. Structured treatment plans referred to individual treatment plans or a stepped care approach. Staff support comprised training of healthcare professionals, supervision, additional staff and expanded prescribing provisions for nurses. Facility reorganisation referred to the physical space at clinics and how this was reorganised to accommodate integrated care.

*Patient involvement in care* was described as patient engagement and self-management support. Patient engagement referred to engaging with patients and considering their views in decision-making and treatment plans. Self-management support included problem-solving, goal setting, self-monitoring and self-care education.

**Fig. 4:**
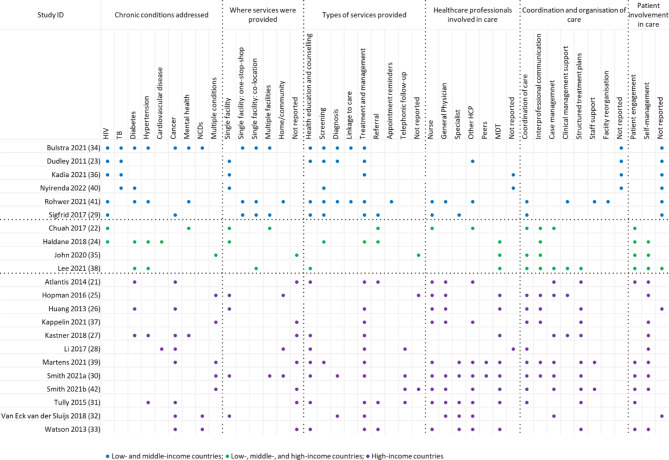
Overview of components and sub-components of integrated models of care

Although components and sub-components differed considerably between and within SRs, we observed some similarities related to where studies included in the SRs were conducted: in LMICs, in LMICs and HICs, or in HIC only. Reviews in each of these categories reported similar subcomponents, whereas we observed differences in the reported sub-components across these categories (Fig. 4).

In the six SRs that only included studies from LMICs [23, 29, 34, 36, 40, 41], integrated models of care were mainly described in terms of the conditions addressed, where services were provided and the types of services that were offered. All but one SR included studies that integrated services for HIV with other conditions, and four of the six studies included studies that integrated services for TB. Three of the SRs in this category also reported that services were offered in a one-stop-shop, a term that was not used for studies conducted in HICs. Services offered mostly included screening and treatment of the condition. SRs in this category also reported on linkage to care, a service that was not reported in SRs that included studies from HICs. Furthermore, only one SR reported that specialists were part of the team providing care, while coordination of care was only reported in two SRs. Patient involvement in care was not reported in any of the SRs that only included studies from LMICs.

The four SRs that included studies from LMICs and HICs generally reported on few subcomponents [22, 24, 35, 38]. All reviews reported on how care was coordinated and interprofessional communication, as well as patient engagement in care.

In the twelve SRs that only included studies from HICs [21, 25–28, 30–33, 37, 39, 42], integrated models of care were generally described in terms of who provided care, how care was coordinated and patient involvement in care. These SRs mainly addressed multiple conditions including, cancer, diabetes, hypertension, mental health and NCDs. Services reported for this category related mostly to health education and counselling and treatment and management of the condition. A wide range of healthcare professionals provided care, and most SRs reported that a multi-disciplinary team was involved in care. SRs in this category described a wide range of sub-components related to how care was coordinated and organised. Indeed, this was the focus of most SRs included in this category. Furthermore, all but two reviews reported on patient involvement in care, and all but three reviews described subcomponents related to self-management.

### Effectiveness of integrated models of care

The findings on the effectiveness of integrated models of care, as reported in the conclusions of included SRs, are summarised in Additional file 5. Reported conclusions on the effectiveness of integrated models of care were heterogenous for health and process outcomes.

Of the SRs that included studies from LMICs, four reported findings on health outcomes. One SR [34] found that integrated care for HIV and other conditions had health benefits for HIV and other conditions. The other three SRs [36, 40, 41] did not find that integrated care improved health outcomes. Three SRs reported findings on process outcomes and reported an increase in the uptake of services for integrated models of care [23, 29, 34].

Two SRs that included studies from both LMICs and HICs, reported improved health outcomes for the patient-centered medical home [35] and interprofessional collaborative practice model [38]. The other two SRs in this category reported that integrated care had positive effects on process outcomes [22, 24].

All SRs that included studies from HICs reported findings on health outcomes. Eight SRs found that collaborative or coordinated care had significant benefits for depression [21, 26–28, 30, 32, 33, 37]. The other four SRs reported that although there might be small improvements in health outcomes [31], the current evidence was insufficient [25] and inconsistent [39], and uncertainties about the effectiveness of interventions for peoples with multi-morbidity remained [42]. Three SRs reported findings on access to health services. One SR reported that integrated models of care may make little or no difference to health services use [30], one SR reported that the intervention significantly increased the use of mental health services [27], and another reported that there was no evidence that comprehensive care reduced the number of primary care visits or costs [25].

## Discussion

We conducted a scoping review to describe the characteristics, components and reported effects of models of integrated care included in systematic reviews on the effectiveness of integrated care for people with multiple chronic conditions. We included 22 SRs investigating various forms of integrated care for a wide range of conditions. Definitions of integrated care varied between SRs. Some referred to integrated care as integration of services, while others only used the terms collaborative or coordinated care. As these models all aimed to treat patients with more than one chronic condition in a more or less integrated manner, we decided within our team, that we would include these under the umbrella term of integrated care.

Integrated models of care were complex and heterogenous, both within and across included SRs, and were poorly reported. Only two SRs used the Template for Intervention Description and Replication (TIDieR) checklist [43] to describe included interventions. This presented a challenge in synthesising and comparing models of care in a meaningful way. However, we identified some similarities in the components of integrated care and summarised these in terms of chronic conditions addressed, where services were provided, the type of services provided, healthcare professionals involved in care, coordination and organisation of care and patient involvement. Furthermore, we identified sub-components within each of the components and described the models of care of included SRs accordingly. Individual SRs did not report on all components and sub-components varied considerably between SRs.

We observed a difference in the reported components and sub-components of interventions based on the income setting of included studies. The different approaches to integrated care in the various income settings might be attributed to the differences in the disease profile of the population and the aims of the intervention. While the main aim in LMICs has been to increase access to care, improve uptake of priority services and increasing efficiency, HICs have focused on shifting care from in-patient care to primary care, and improving quality of care [44].

Our findings regarding heterogeneity in the definition, complexity and context-specific nature of integrated models of care resonate with previous findings [13, 16]. The WHO framework on integrated, people-centered health services [14] recognises the complexity of integrated models of care and proposes five interdependent strategies that should be adopted in a context-sensitive manner, rather than used as a static framework. These are (1) empowering and engaging people and communities, (2) strengthening governance and accountability, (3) reorienting the model of care, (4) coordinating services within and across sectors, and (5) creating an enabling environment. The components that we identified describe integrated care at the level of service delivery and fit under strategy 1 (involvement of patients in care), strategy 3 (conditions, where services were provided, types of services provided, health professionals involved in care) and strategy 4 (coordination and organisation of care).

A recent scoping review that summarised the characteristics of integrated care for NCDs and mental health in LMICs [45] also found that models of care were complex and heterogenous. Authors described the models of care according to pre-specified dimensions, of which the following mirror components that we identified: the condition, type of service, health care provider and health system level (where care was provided).

It is important to have a comprehensive understanding of the components of models of care when planning, implementing and evaluating integrated care interventions, and to report the approaches in a transparent way. Unique aspects linked to the management of specific diseases makes it difficult to synthesise evidence on a broad range of conditions.

We did not aim to evaluate the effectiveness of integrated models of care in this scoping review but extracted a verbatim summary of conclusions of included SRs, which provide an indication of the potential benefit and direction of effects of integrated care and its individual components, both in terms of health and process outcomes. However, this needs to be interpreted with caution, since we did not assess the quality and did not evaluate findings of included SRs. Other studies have found that provision of integrated care has potential benefits on health systems, notable —addressing poor care coordination which is often the main problem cited by patients with multi-morbidity when describing their experiences of health and social care services [46]. Additionally, the literature has suggested that integrated care has a positive effect on the quality [8, 47] and efficiency [24, 48, 49] of services. However, there is still uncertainty about which model of integrated care is most effective, for which conditions, and how these models should be implemented alongside persistent questions over whether the aims of integration are ultimately achievable in any meaningful way [50].

### Strengths and limitations

We followed a rigorous and systematic process to conduct our scoping review. We pre-specified our eligibility criteria and conducted a comprehensive search to identify the available evidence. To minimise bias, two authors independently selected studies and extracted data.

We set out to conduct an overview of SRs, focussing on the effectiveness of integration of care. However, during study selection and data extraction, we realised that included interventions were very complex and heterogenous, which made it difficult to compare models of care across SR. Furthermore, we found that we needed a better understanding of the available models of care before evaluating their effectiveness. We therefore revised our strategy to conduct a scoping review, focussing on the characteristics of various models of integrated care as a first step. Although we provide a summary of verbatim conclusions from included SRs, this needs to be interpreted with caution, as we did not assess the quality of included SRs.

Even though our search was comprehensive, we acknowledge that including a wider range of studies and not restricting it to SRs would have been useful to get a good understanding of integrated models of care. Our findings are based on how authors of included SRs reported the interventions and we did not review primary studies. Poor reporting of primary studies in SRs was limiting.

Our logic model describing integrated models of care (Fig. 1) depicts our conceptualisation of fully integrated models of care and partially integrated models of care, which mainly refers to the services that are being offered, ideally in a one-stop-shop setting. However, we found that it was difficult to categorise models of care using these categories, mainly because services offered as part of the interventions were poorly described. If reported, most SRs only described services offered for one condition, which we assumed to be the ‘add-on’ condition. The other components of integrated care that we described in our scoping review are not depicted in the logic model. Our model might be too simplistic to describe the range of interventions considered as part of integrated care in various contexts and health systems. An expansion of this model is therefore warranted.

## Conclusion

Integrated models of care were heterogenous within and across included SRs. Although there was a lack of a common definition of integrated care, there were some common components of integrated care reported across included SRs. We observed a difference in the conceptualisation and description of integrated care according to the income setting of the included studies, and information that allows the identification of effective components of integrated care was lacking. There is a need to develop a structured framework to compare the effectiveness of integrated models of care that can be used in future primary research studies and evidence syntheses. Detailed, standardised and transparent reporting of the intervention components and their effectiveness on patient-relevant and health system outcomes is needed. We encourage authors of primary and secondary research to use the TIDieR checklist when reporting on integrated models of care.

### Electronic supplementary material

Below is the link to the electronic supplementary material.

Supplementary Material 1

Supplementary Material 2

Supplementary Material 3

Supplementary Material 4

Supplementary Material 5

Supplementary Material 6

Supplementary Material 7

## Data Availability

All data generated or analysed during this study are included in this published article and its supplementary information files.

## Abbreviations

BMBF: German Federal Ministry of Education and Research (Bundesministerium für Bildung und Forschung)
CEBHA+: Collaboration for Evidence-based Healthcare and Public Health in Africa
HICs: High income countries
HIV: Human immunodeficiency virus
LMICs: Low - Middle- Income Countries
NCDs: Non-communicable diseases
PHC: Primary Healthcare
PRISMA: Preferred Reporting Items for Systematic Reviews and Meta-Analyses
PROSPERO: Prospective Register of Systematic Reviews
RCTs: Randomised controlled trials
SRs: Systematic Reviews
TB: Tuberculosis
TIDieR: Template for Intervention Description and Replication
WHO: World Health Organisation
