# Supplementary material for: Models of integrated care for multi-morbidity assessed in systematic reviews: a scoping review
Source: BMC Health Serv Res. 2023 Aug 23;23:894. doi: 10.1186/s12913-023-09894-7 (PMC10463690; doi:10.1186/s12913-023-09894-7)
Supplement: Supplementary file 1 — Supplementary Material 1 [file 12913_2023_9894_MOESM1_ESM.pdf]

Additional file 1: Search strategies for electronic databases

Embase

1 integrated health care system/ or integrated health care.mp.

2 *patient care/

3 ("comprehensive healthcare" or "comprehensive care" or "Continuity of Patient Care" or "continuity of care" or "continuity of healthcare" or "Patient-Centered Care").ti.

4 ("comprehensive healthcare" or "comprehensive care" or "Continuity of Patient Care" or "continuity of care" or "continuity of healthcare" or "Patient-Centered Care").ab.

5 (referral and consultation).mp. [mp=title, abstract, heading word, drug trade name, original title, device manufacturer, drug manufacturer, device trade name, keyword, floating subheading word, candidate term word]

6 ((integrated or integration) adj2 (care or services or program* or delivery or management)).ab.

7 ((integrated or integration) adj2 (care or services or program* or delivery or management)).ti.

8 ((coordination or coordinated) adj2 (care or services or program* or delivery or management)).ti.

9 ((coordination or coordinated) adj2 (care or services or program* or delivery or management)).ab.

10 ((horizontal or vertical) adj2 (care or services or program* or delivery or management)).ab.

11 ((horizontal or vertical) adj2 (care or services or program* or delivery or management)).ti.

12 (Multiteam or multi-team or multi-care or multicare or multiclinic or multiservice or multi-program* or multidelivery or multi-management).ti. or (Multiteam or multi-team or multi-care or multicare or multiclinic or multiservice or multi-program* or multidelivery or multi-management).ab.

13 *health care delivery/

14 (delivery adj2 healthcare).mp. [mp=title, abstract, heading word, drug trade name, original title, device manufacturer, drug manufacturer, device trade name, keyword, floating subheading word, candidate term word]

15 1 or 2 or 3 or 4 or 5 or 6 or 7 or 8 or 9 or 10 or 11 or 12 or 13 or 14

16 systematic review.mp. or "systematic review"/

17 meta analysis/

18 ("pooled data" or "pooling data" or "pooled data" or "pooled results").mp. [mp=title, abstract, heading word, drug trade name, original title, device manufacturer, drug manufacturer, device trade name, keyword, floating subheading word, candidate term word]

19 (metaanalysis or meta-analysis).ti. or (metaanalysis or meta-analysis).ab.

20 ("research synthesis" or "synthesized research").ti. or ("research synthesis" or "synthesized research").ab.

21 16 or 17 or 18 or 19 or 20

22 15 and 21

23 hypertension.mp. or *hypertension/

24 (hypertension or hypertention or "blood pressure" or "arterial pressure" or systolic or diastolic).ti. or (hypertension or hypertention or "blood pressure" or "arterial pressure" or systolic or diastolic).ab.

25 diabetes.mp. or diabetes mellitus/

26 exp Neoplasms/

27 cardiovascular disease/

28 "heart disease".ti. or "heart disease*".ab.

29 *kidney disease/

30 ("kidney failure" or "renal failure" or "chronic kidney disease" or "renal disease").ti. or ("kidney failure" or "renal failure" or "chronic kidney disease" or "renal disease").ab.

31 (dyslipidaemia or dyslipidemia or cholesterol or LDL or HDL or triglyceride or triglycerides or low density lipoprotein or high density lipoprotein or low-density lipoprotein or high-density lipoprotein).ti. or (dyslipidaemia or dyslipidemia or cholesterol or LDL or HDL or triglyceride or triglycerides or low density lipoprotein or high density lipoprotein or low-density lipoprotein or high-density lipoprotein).ab.

32 HIV infection.mp. or Human immunodeficiency virus infection/

33 tuberculosis/

34 non-communicable diseases.mp. or non communicable disease/

35 comorbidity.mp. or comorbidity/

36 multimorbidity.mp. or multiple chronic conditions/

37 (multi-disease* or multidisease* or multi disease* or multiple condition* or multi-condition* or multi condition* or multiple illness* or multi-illness* or multi illness* or multiple syndrome* or multi-syndrome* or multi syndrome* or concurrent condition* or concurrent illness* or concurrent disease* or co-existing disease* or coexisting disease* or co-existing illness* or coexisting illness* or co-existing syndrome* or coexisting syndrome* or co-existing condition* or coexisting condition* or co-occurring disease* or co occuring disease* or cooccuring disease* or co-occurring illness* or co occurring illness* or cooccurring illness* or co-occurring syndrome* or co occurring syndrome* or cooccurring syndrome* or co-occurring condition* or co occurring condition* or cooccurring condition*).ti.

38 (multi-disease* or multidisease* or multi disease* or multiple condition* or multi-condition* or multi condition* or multiple illness* or multi-illness* or multi illness* or multiple syndrome* or multi-syndrome* or multi syndrome* or concurrent condition* or concurrent illness* or concurrent disease* or co-existing disease* or coexisting disease* or co-existing illness* or coexisting illness* or co-existing syndrome* or coexisting syndrome* or co-existing condition* or coexisting condition* or co-occurring disease* or co occuring disease* or cooccuring disease* or co-occurring illness* or co occurring illness* or cooccurring illness* or co-occurring syndrome* or co occurring syndrome* or cooccurring syndrome* or co-occurring condition* or co occurring condition* or cooccurring condition*).ab.

39 (chronic disease* or lifestyle disease*).mp. [mp=title, abstract, heading word, drug trade name, original title, device manufacturer, drug manufacturer, device trade name, keyword, floating subheading word, candidate term word]

40 23 or 24 or 25 or 26 or 27 or 28 or 29 or 30 or 31 or 32 or 33 or 34 or 35 or 36 or 37 or 38 or 39

Medline PubMed

Search	Query
#28	Search **((#8 AND #13 AND #26))** Filters: **Publication date from 2018/11/01 to 2020/02/10**
#27	Search **(#8 AND #13 AND #26)**
#26	Search **(#14 OR #15 OR #16 OR #17 OR #18 OR #19 OR #20 OR #21 OR #22 OR #23 OR #24 OR #25)**
#25	Search **(multi-disease* OR multidisease* OR multi disease* OR multiple condition* OR multi-condition* OR multi condition* OR multiple illness* OR multi-illness* OR multi illness* OR multiple syndrome* OR multi-syndrome* OR multi syndrome* OR concurrent condition* OR concurrent illness* OR concurrent disease* OR co-existing disease* OR coexisting disease* OR co-existing illness* OR coexisting illness* OR co-existing syndrome* OR coexisting syndrome* OR co-existing condition* OR coexisting condition* OR co-occurring disease* OR co occuring disease* OR cooccuring disease* OR co-occurring illness* OR co occurring illness* OR cooccurring illness* OR co-occurring syndrome* OR co occurring syndrome* OR cooccurring syndrome* OR co-occurring condition* OR co occurring condition* OR cooccurring condition*)**
#24	Search **(chronic disease* OR lifestyle disease* OR "diseases of lifestyle" OR "disease of lifestyle" OR "Multiple Chronic Conditions"[Mesh] OR "Chronic Disease"[Mesh])**
#23	Search **((comorbid* OR co-morbid* OR "co morbidity" OR multimorbidity OR multi-morbid OR "multi morbidity")[title/abstract] OR "Multimorbidity"[Mesh] OR "Comorbidity"[Mesh])**
#22	Search **("noncommunicable disease" OR "noncommunicable diseases" OR "non-communicable disease" OR "non-communicable diseases" OR NCD OR NCDs OR "Noncommunicable Diseases"[Mesh])**
#21	Search **("tuberculosis"[Mesh]) OR ((tuberculosis[Title/Abstract] OR tuberculoses[Title/Abstract] OR tb)[Title/Abstract])**

Search	Query
#20	Search **(((((HIV OR hiv-1 OR hiv-2* OR hiv1 OR hiv2 OR hiv infect* OR human immunodeficiency virus OR human immune deficiency virus OR human immuno-deficiency virus OR human immune-deficiency virus OR ((human immun*) AND (deficiency virus)) OR acquired immunodeficiency syndromes OR acquired immune deficiency syndrome OR acquired immuno-deficiency syndrome OR acquired immune-deficiency syndrome OR ((acquired immun*) AND (deficiency syndrome)) OR HIV/AIDS)))) OR ((HIV infections [MeSH] OR HIV [MeSH])))**
#19	Search **((dyslipidaemia OR dyslipidemia OR cholesterol OR LDL OR HDL OR triglyceride OR triglycerides OR low density lipoprotein OR high density lipoprotein OR low-density lipoprotein OR high-density lipoprotein)[title/abstract] OR "Dyslipidemias"[Mesh])**
#18	Search **(("Kidney Diseases"[Mesh] OR "kidney disease" OR "kidney failure" OR "renal failure" OR "chronic kidney disease" OR "renal disease"))**
#17	Search **(("heart disease" OR cardiovascular OR "Cardiovascular Diseases"[Mesh]))**
#16	Search **("Neoplasms"[Mesh]) OR cancer[Title/Abstract]**
#15	Search **(("Diabetes Mellitus"[Mesh]) OR "diabetes mellitus"[Title/Abstract]) OR diabetes[Title/Abstract]**
#14	Search **("Hypertension"[Mesh]) OR ((hypertension[Title/Abstract] OR hypertention[Title/Abstract] OR "blood pressure"[Title/Abstract] OR "arterial pressure"[Title/Abstract] OR systolic[Title/Abstract] OR diastolic)[[Title/Abstract])**
#13	Search **(#9 OR #10 OR #11 OR #12)**
#12	Search **("Meta-Analysis" [Publication Type] OR "Network Meta-Analysis"[Mesh])**
#11	Search **(“pooled data” OR “pooling data” OR “pooled data” OR “pooled results”)**
#10	Search **(("synthesised research"[Title/Abstract]) OR "synthesized research"[Title/Abstract]) OR "research synthesis"[Title/Abstract]**

Search	Query
#9	Search **("systematic review"[Title/Abstract]) OR meta-analysis[Title/Abstract]**
#8	Search **(#1 OR #2 OR #3 OR #4 OR #5 OR #6 OR #7)**
#7	Search **(“multi team” OR multiteam “multi care” OR multicare OR “multi clinic” OR multiclinic OR “multi service” OR multiservice OR “multi program” OR multiprogram OR “multi programme” OR “multi delivery” OR multidelivery OR “multi management”)**
#6	Search **(horizontal care OR vertical care OR horizontal services OR vertical services OR horizontal programmes OR horizontal programs OR vertical programmes OR vertical programs OR horizontal service delivery OR vertical service delivery OR horizontal services OR vertical services OR horizontal delivery OR vertical management OR vertical management)**
#5	Search **(co-ordinat* care OR "co-ordination of care" OR co-ordinat* services OR "co-ordination of services" OR co-ordinat* programmes OR co-ordinat* programs OR “co-ordination of programmes” OR “co-ordination of programs” OR co-ordinat* service delivery OR “co-ordination of service delivery” OR co-ordinat* services OR “co-ordination of services” OR co-ordinat* delivery OR co-ordinat* management OR “co-ordination of management”)**
#4	Search **(coordinat* care OR "coordination of care" OR coordinat* services OR "coordination of services" OR coordinat* programmes OR coordinat* programs OR “coordination of programmes” OR “coordination of programs” OR coordinat* service delivery OR “coordination of service delivery” OR coordinat* services OR “coordination of services” OR coordinat* delivery OR coordinat* management OR “coordination of management”)**
#3	Search **(integrat* care OR "integration of care" OR integrat* services OR "integration of services" OR integrat* programmes OR integrat* programs OR “integration of programmes” OR “integration of programs” OR integrat* service delivery OR “integration of service delivery” OR integrat* services OR “integration of services” OR integrat* delivery OR integrat* management OR “integration of management”)**
#2	Search **("Referral and Consultation"[Mesh] OR (referral AND consultation))**

Search	Query
#1	Search **("Delivery of Health Care, Integrated"[Mesh] OR “delivery of care” OR “delivery of health” OR "delivery of healthcare" OR "Comprehensive Health Care"[Mesh] OR "comprehensive healthcare" OR "comprehensive care" OR "comprehensive health" OR "Continuity of Patient Care"[Mesh] OR "continuity of patient care" OR "continuity of care" OR "continuity of health" OR "continuity of healthcare" OR "Patient-Centered Care"[Mesh] OR "patient centered care" OR "patient centred care")**

Cochrane Database of Systematic Reviews

#1 MeSH descriptor: [Delivery of Health Care, Integrated] explode all trees

#2 MeSH descriptor: [Comprehensive Health Care] explode all trees

#3 MeSH descriptor: [Continuity of Patient Care] explode all trees

#4 MeSH descriptor: [Patient-Centered Care] explode all trees

#5 “delivery of care” OR “delivery of health” OR "delivery of healthcare" OR "comprehensive healthcare" OR "comprehensive care" OR "comprehensive health" OR "continuity of patient care" OR "continuity of care" OR "continuity of health" OR "continuity of healthcare" OR "patient centered care" OR "patient centred care"

#6 #1 OR #2 OR #3 OR #4 OR #5

#7 MeSH descriptor: [Referral and Consultation] explode all trees

#8 referral AND consultation

#9 #7 OR #8

#10 "integrating care" OR "integration of care" OR "integrated care" OR "integrating services" OR "integration of services" OR "integrated services" OR "integrating programmes" OR "integrating programs" OR “integration of programmes” OR “integration of programs” OR "integrated programmes" OR "integrated programs" OR "integrating service delivery" OR "integrated service delivery" OR “integration of service delivery” OR "integrating services" OR “integration of services” OR "integrated services" OR "integrated delivery" OR "integrated management" OR “integration of management”

#11 "coordinating care" OR "coordinated care" OR "coordination of care" OR "coordinating services" OR "coordination of services" OR "coordinated services" OR "coordinating programmes" OR "coordinating programs" OR “coordination of programmes” OR "coordinated programmes" OR "coordinated programs" OR “coordination of programs” OR "coordinating service delivery" OR “coordination of service delivery” OR "coordinated service delivery" OR "coordinated services" OR “coordination of services” OR "coordinated delivery" OR "coordinating management" OR "coordinated management" OR “coordination of management”

#12 "co-ordinating care" OR "co-ordinated care" OR "co-ordination of care" OR "co-ordinating services" OR "co-ordination of services" OR "co-ordinated services" OR "co-ordinating programmes" OR "co-ordinating programs" OR “co-ordination of programmes” OR "co-ordinated

programmes" OR "co-ordinated programs" OR “co-ordination of programs” OR "co-ordinating service delivery" OR “co-ordination of service delivery” OR "co-ordinated service delivery" OR "co-ordinated services" OR “co-ordination of services” OR "co-ordinated delivery" OR "co-ordinating management" OR "co-ordinated management" OR “co-ordination of management”

#13 "horizontal care" OR "vertical care" OR "horizontal services" OR "vertical services" OR "horizontal programmes" OR "horizontal programs" OR "vertical programmes" OR "vertical programs" OR "horizontal service delivery" OR "vertical service delivery" OR "horizontal services" OR "vertical services" OR "horizontal delivery" OR "vertical management" OR "vertical management"

#14 “multi team” OR multiteam “multi care” OR multicare OR “multi clinic” OR multiclinic OR “multi service” OR multiservice OR “multi program” OR multiprogram OR “multi programme” OR “multi delivery” OR multidelivery OR “multi management” OR multi-team OR multi-care OR multi-clinic OR multi-service* OR multi-program* OR multi-programme* OR multi-delivery OR multi-management

#15 #6 OR #9 OR #10 OR #11 OR #12 OR #13 OR #14

#16 MeSH descriptor: [Hypertension] explode all trees

#17 hypertension OR hypertention OR "blood pressure" OR “arterial pressure” OR systolic OR diastolic

#18 MeSH descriptor: [Diabetes Mellitus] explode all trees

#19 diabetes OR "diabetes mellitus"

#20 MeSH descriptor: [Neoplasms] explode all trees

#21 cancer

#22 MeSH descriptor: [Cardiovascular Diseases] explode all trees

#23 "heart disease" OR cardiovascular

#24 MeSH descriptor: [Kidney Diseases] explode all trees

#25 "kidney disease" OR "kidney failure" OR "renal failure" OR "chronic kidney disease" OR "renal disease"

#26 MeSH descriptor: [Dyslipidemias] explode all trees

#27 dyslipidaemia OR dyslipidemia OR cholesterol OR LDL OR HDL OR triglyceride OR triglycerides OR "low density lipoprotein" OR "high density lipoprotein" OR "low-density lipoprotein" OR "high-density lipoprotein"

#28 MeSH descriptor: [HIV] explode all trees

#29 MeSH descriptor: [HIV Infections] explode all trees

#30 HIV OR hiv-1 OR hiv-2* OR hiv1 OR hiv2 OR "hiv infection" OR "hiv infections" OR "human immunodeficiency virus" OR "human immune deficiency virus" OR "human immuno-deficiency virus" OR "human immune-deficiency virus"

#31 (human immun*) AND (deficiency virus)

#32 acquired immunodeficiency syndromes OR acquired immune deficiency syndrome OR acquired immuno-deficiency syndrome OR acquired immune-deficiency syndrome

#33 (acquired immun*) AND (deficiency syndrome)

#34 HIVAIDS

#35 MeSH descriptor: [Tuberculosis] explode all trees

#36 tuberculosis OR tuberculoses OR tb

#37 MeSH descriptor: [Noncommunicable Diseases] explode all trees

#38 "noncommunicable disease" OR "noncommunicable diseases" OR "non-communicable disease" OR "non-communicable diseases" OR NCD OR NCDs

#39 MeSH descriptor: [Comorbidity] explode all trees

#40 MeSH descriptor: [Multimorbidity] explode all trees

#41 comorbid* OR co-morbid* OR "co morbidity" OR multimorbidity OR multi-morbid OR "multi morbidity"

#42 “multi-disease” OR multidisease* OR “multi disease” OR “multiple condition” OR multi-condition* OR “multi condition” OR “multiple illness” OR multi-illness* OR “multi illness” OR “multiple syndrome” OR multi-syndrome* OR “multi syndrome” OR “concurrent condition” OR “concurrent illness” OR “concurrent disease” OR co-existing disease* OR “coexisting disease” OR co-existing illness* OR “coexisting illness” OR co-existing syndrome* OR “coexisting syndrome” OR co-existing condition* OR “coexisting condition” OR co-occurring disease* OR “co occuring disease” OR “cooccuring disease” OR co-occurring illness* OR “co occurring illness” OR “cooccurring illness” OR co-occurring syndrome* OR “co occurring syndrome” OR “cooccurring syndrome” OR co-occurring condition* OR “co occurring condition” OR “cooccurring condition”

#43 MeSH descriptor: [Chronic Disease] explode all trees

#44 MeSH descriptor: [Multiple Chronic Conditions] explode all trees

#45 "chronic disease" OR "lifestyle disease" OR "diseases of lifestyle" OR "disease of lifestyle"

#46 #16 OR #17 OR #18 OR #19 OR #20 OR #21 OR #22 OR #23 OR #24 OR #25 OR #26 OR #27 OR #28 OR #29 OR #30 OR #31 OR #32 OR #33 OR #34 OR #35 OR #36 OR #37 OR #38 OR #39 OR #40 OR #41 OR #42 OR #43 OR #44 OR #45

Epistemonikos

(title:("comprehensive care" OR "continuity of care" OR "integrated care" OR "coordinated care" OR "integrated services" OR "comprehensive services" OR "coordinated services" OR "integrated programmes" OR "integrated programs") OR abstract:("comprehensive care" OR "continuity of care" OR "integrated care" OR "coordinated care" OR "integrated services" OR "comprehensive services" OR "coordinated services" OR "integrated programmes" OR "integrated programs")) AND (title:(Hypertension OR "high blood pressure" OR diabetes OR cancer OR neoplasms OR "cardiovascular disease" OR "cardiovascular diseases" OR "heart disease" OR "kidney disease" OR "renal disease" OR dyslipidaemia OR dyslipidemia OR cholesterol OR "density lipoprotein" OR HDL OR LDL OR HIV OR "human immunodeficiency virus" OR AIDS OR TB OR tuberculosis OR NCDs OR NCD OR "non-communicable disease" OR comborbidity OR multimorbidity OR co-morbidity OR multi-morbidity OR "chronic disease" OR "lifestyle disease" OR "disease of lifestyle") OR abstract:(Hypertension OR "high blood pressure" OR diabetes OR cancer OR neoplasms OR "cardiovascular disease" OR "cardiovascular diseases" OR "heart disease" OR "kidney disease" OR "renal disease" OR dyslipidaemia OR dyslipidemia OR cholesterol OR "density lipoprotein" OR HDL OR LDL OR HIV OR "human immunodeficiency virus" OR AIDS OR TB OR tuberculosis OR NCDs OR NCD OR "non-communicable disease" OR comborbidity OR multimorbidity OR co-morbidity OR multi-morbidity OR "chronic disease" OR "lifestyle disease" OR "disease of lifestyle"))

AND

(title:("comprehensive care" OR "continuity of care" OR "integrated care" OR "coordinated care" OR "integrated services" OR "comprehensive services" OR "coordinated services" OR "integrated programmes" OR "integrated programs") OR abstract:("comprehensive care" OR "continuity of care" OR "integrated care" OR "coordinated care" OR "integrated services" OR "comprehensive services" OR "coordinated services" OR "integrated programmes" OR "integrated programs"))

Healthsystemsevidence.org (https://www.healthsystemsevidence.org/?lang=en)

multimorbidity OR comorbidity AND "continuity of care" OR "integrated care"
