# Supplementary material for: Models of integrated care for multi-morbidity assessed in systematic reviews: a scoping review
Source: BMC Health Serv Res. 2023 Aug 23;23:894. doi: 10.1186/s12913-023-09894-7 (PMC10463690; doi:10.1186/s12913-023-09894-7)
Supplement: Supplementary file 2 — Supplementary Material 2 [file 12913_2023_9894_MOESM2_ESM.pdf]

Additional file 2: Excluded studies

Study ID	Reason for exclusion
1. Abrams, Watkins et al. (2020)	Wrong population
2. Alyousef, Naughton et al. (2021)	Wrong intervention
3. Bartolo, Pacheco et al. (2019)	Wrong population
4. Brighton, Miller et al. (2019)	Wrong study design
5. Busetto, Luijkx et al. (2016)	Wrong population
6. Chang, Knobf et al. (2019)	Wrong intervention
7. Cimpean and Drake (2011)	Wrong study design (no risk of bias assessment)
8. Cui, Dong et al. (2019)	Wrong population
9. Davidson, Kelly et al. (2022)	Wrong question type
10. Deschodt, Laurent et al. (2020)	Wrong population
11. Flood, Hane et al. (2020)	Wrong population
12. Godinho, Jonnagaddala et al. (2020)	Wrong intervention
13. Green (2013)	Wrong study design
14. Hendriks, Gallagher et al. (2017)	Wrong population
15. Hudson, Rutherford et al. (2018)	Wrong setting
16. Hully, Mallah et al. (2022)	Wrong intervention
17. Kemp, Weiner et al. (2018)	Wrong question type
18. Leach, Eaton et al. (2019)	Wrong population
19. Legido-Quigley, Montgomery et al. (2013)	Wrong question type
20. Lemmens, Nieboer et al. (2009)	Wrong population
21. Lindegren, Kennedy et al. (2012)	Wrong population
22. Lores, Goess et al. (2019)	Wrong setting
23. Lv, Kringle et al. (2022)	Wrong intervention
24. McCarter, Britton et al. (2018)	Wrong setting
25. Medley, Bachanas et al. (2015)	Wrong population
26. Miller, Brighton et al. (2018)	Wrong population
27. Muhula, Gachohi et al. (2022)	Wrong population
28. Nkhoma, Sitali et al. (2022)	Wrong intervention
29. Nugent, Barnabas et al. (2018)	Wrong question type
30. Okusanya, Kimaru et al. (2022)	Wrong population
31. O'Neil, Sanderson et al. (2011)	Wrong intervention
32. Osborn, Demoncada et al. (2006)	Wrong intervention
33. O'Shea, Ledwidge et al. (2022)	Wrong intervention
34. Ouwens, Hulscher et al. (2009)	Wrong population
35. Panagioti, Bower et al. (2016)	Wrong study design
36. Patel and Jafferany (2020)	Wrong question type
37. Patel, Rose et al. (2018)	Wrong question type
38. Poot, Meijer et al. (2021)	Wrong population

39. Robb and Hanson-Abromeit (2014)	Wrong population
40. Robertson, Baines et al. (2017)	Wrong intervention
41. Rutledge, Redwine et al. (2013)	Wrong intervention
42. Sabri and Gielen (2019)	Wrong intervention
43. Savage, Hegarty et al. (2016)	Wrong population
44. Shaw, Sethi et al. (2019)	Wrong question type
45. Smith, Soubhi et al. (2012)	Previous version of included review
46. Socías, Karamouzian et al. (2019)	Wrong population
47. Thombs, de Jonge et al. (2008)	Wrong study design (no risk of bias assessment)
48. Uyei, Coetzee et al. (2011)	Wrong study design (no risk of bias assessment)
49. Vermunt, Harmsen et al. (2017)	Wrong intervention
50. Zhao, Ma et al. (2022)	Wrong population

References

Abrams, J., D. A. Watkins, L. H. Abdullahi, L. J. Zuhlke and M. E. Engel (2020). "Integrating the prevention and control of rheumatic heart disease into country health systems: A systematic review and meta-analysis." Global Heart **15**(1): 62.

Alyousef, M., C. Naughton, C. Bradley and E. Savage (2021). "Primary healthcare reform for chronic conditions in countries with high or very high human development index: A systematic review." Chronic Illn: 17423953211059143.

Bartolo, A., E. Pacheco, F. Rodrigues, A. Pereira, S. Monteiro and I. M. Santos (2019). "Effectiveness of psycho-educational interventions with telecommunication technologies on emotional distress and quality of life of adult cancer patients: a systematic review." Disability and rehabilitation **41**(8): 870-878.

Brighton, L. J., S. Miller, M. Farquhar, S. Booth, D. Yi, W. Gao, S. Bajwah, W. D. Man, I. J. Higginson and M. Maddocks (2019). "Holistic services for people with advanced disease and chronic breathlessness: a systematic review and meta-analysis." Thorax **74**(3): 270-281.

Busetto, L., K. G. Luijkx, A. M. J. Elissen and H. J. M. Vrijhoef (2016). "Intervention types and outcomes of integrated care for diabetes mellitus type 2: A systematic review." Journal of Evaluation in Clinical Practice **22**(3): 299-310.

Chang, P. S., T. Knobf, B. Oh and M. Funk (2019). "Physical and Psychological Health Outcomes of Qigong Exercise in Older Adults: A Systematic Review and Meta-Analysis." The American journal of Chinese medicine **47**(2): 301-322.

Cimpean, D. and R. E. Drake (2011). "Treating co-morbid chronic medical conditions and anxiety/depression." Epidemiology and psychiatric sciences **20**(2): 141-150.

Cui, X., W. Dong, H. Zheng and H. Li (2019). "Collaborative care intervention for patients with chronic heart failure: A systematic review and meta-analysis." Medicine **98**(13): e14867.

Davidson, A. R., J. Kelly, L. Ball, M. Morgan and D. P. Reidlinger (2022). "What do patients experience? Interprofessional collaborative practice for chronic conditions in primary care: an integrative review." BMC primary care **23**(1): 8.

Deschodt, M., G. Laurent, L. Cornelissen, O. Yip, F. Zúñiga, K. Denhaerynck, M. Briel, A. Karabegovic and S. De Geest (2020). "Core components and impact of nurse-led integrated care models for home-dwelling older people: A systematic review and meta-analysis." Int J Nurs Stud **105**: 103552.

Flood, D., J. Hane, M. Dunn, S. J. Brown, B. H. Wagenaar, E. A. Rogers, M. Heisler, P. Rohloff and V. Chopra (2020). "Health system interventions for adults with type 2 diabetes in low- And middle-income countries: A systematic review and meta-analysis." PLoS Medicine **17**(11): e1003434.

Godinho, M. A., J. Jonnagaddala, N. Gudi, R. Islam, P. Narasimhan and S. T. Liaw (2020). "mHealth for Integrated People-Centred Health Services in the Western Pacific: A Systematic Review." International Journal of Medical Informatics **142**: 104259.

Green, B. B. (2013). "Caring for patients with multiple chronic conditions: Balancing evidenced-based and patient-centered care." Journal of the American Board of Family Medicine **26**(5): 484-485.

Hendriks, J. M. L., C. Gallagher, R. Mahajan, A. Elliott, M. E. Middeldorp, D. Lau and P. Sanders (2017). "Integrated Care Management in Atrial Fibrillation is associated with improved outcomes - a systematic review and meta-analysis." International Journal of Integrated Care (IJIC) **17**(3): 196-197.

Hudson, M., G. W. Rutherford, S. Weiser and E. Fair (2018). "Linking private, for-profit providers to public sector services for HIV and tuberculosis co-infected patients: A systematic review." PloS one **13**(4): e0194960.

Hully, A., R. Mallah, G. Villa and Y. Gilleece (2022). "Integrating services to improve quality of care for women living with HIV: A global systematic review." HIV Med **23**(4): 310-318.

Kemp, C. G., B. J. Weiner, K. H. Sherr, L. E. Kupfer, P. K. Cherutich, D. Wilson, E. H. Geng and J. N. Wasserheit (2018). "Implementation science for integration of HIV and non-communicable disease services in sub-Saharan Africa: A systematic review." AIDS **32**(Supplement 1): S93-S105.

Leach, M. J., H. Eaton, T. Agnew, M. Thakkar and M. Wiese (2019). "The effectiveness of integrative healthcare for chronic disease: A systematic review." International journal of clinical practice **73**(4): e13321.

Legido-Quigley, H., C. M. Montgomery, P. Khan, R. Atun, A. Fakoya, H. Getahun and A. D. Grant (2013). "Integrating tuberculosis and HIV services in low- and middle-income countries: A systematic review." Tropical Medicine and International Health **18**(2): 199-211.

Lemmens, K. M., A. P. Nieboer and R. Huijsman (2009). "A systematic review of integrated use of disease-management interventions in asthma and COPD." Respir Med **103**(5): 670-691.

Lindegren, M. L., C. E. Kennedy, D. Bain-Brickley, H. Azman, A. A. Creanga, L. M. Butler, A. B. Spaulding, T. Horvath and G. E. Kennedy (2012). "Integration of HIV/AIDS services with maternal, neonatal and child health, nutrition, and family planning services." Cochrane database of systematic reviews (Online) **9**: CD010119.

Lores, T. J., C. Goess, A. Mikocka-Walus, K. Collins, A. Burke, A. Chur-Hansen, P. Delfabbro and J. M. Andrews (2019). "HEALTHCARE UTILISATION COSTS MAY BE REDUCED BY INTEGRATING PSYCHOLOGICAL CARE INTO IBD MANAGEMENT." Gastroenterology **156**(6 Supplement 1): S-834.

Lv, N., E. A. Kringle and J. Ma (2022). "Integrated Behavioral Interventions for Adults with Comorbid Obesity and Depression: a Systematic Review." Curr Diab Rep **22**(4): 157-168.

McCarter, K., B. Britton, A. L. Baker, S. A. Halpin, A. K. Beck, G. Carter, C. Wratten, J. Bauer, E. Forbes, D. Booth and L. Wolfenden (2018). "Interventions to improve screening and appropriate referral of patients with cancer for psychosocial distress: systematic review." BMJ open **8**(1): e017959.

Medley, A., P. Bachanas, M. Grillo, N. Hasen and U. Amanyeiwe (2015). "Integrating prevention interventions for people living with HIV into care and treatment programs: a systematic review of the evidence." J Acquir Immune Defic Syndr **68 Suppl 3**(0 3): S286-296.

Miller, S., L. Brighton, M. Farquhar, S. Booth, D. Yi, W. Gao, S. Bajwah, W. D. C. Man, I. J. Higginson and M. Maddocks (2018). "Holistic services for chronic breathlessness in people with advanced disease: A systematic review and meta-analysis." European Respiratory Journal **52**(Supplement 62).

Muhula, S., J. Gachohi, Y. Kombe and S. Karanja (2022). "Interventions to improve early retention of patients in antiretroviral therapy programmes in sub-Saharan Africa: A systematic review." PLoS ONE **17**(2): e0263663.

Nkhoma, L., D. C. Sitali and J. M. Zulu (2022). "Integration of family planning into HIV services: a systematic review." Annals of Medicine **54**(1): 393-403.

Nugent, R., R. V. Barnabas, I. Golovaty, B. Osetinsky, D. A. Roberts, C. Bisson, L. Courtney, P. Patel, G. Yonga and D. Watkins (2018). "Costs and cost-effectiveness of HIV/noncommunicable disease integration in Africa: From theory to practice." AIDS **32**(Supplement 1): S83-S92.

O'Neil, A., K. Sanderson, B. Oldenburg and C. B. Taylor (2011). "Impact of depression treatment on mental and physical health-related quality of life of cardiac patients: a meta-analysis." Journal of cardiopulmonary rehabilitation and prevention **31**(3): 146-156.

O'Shea, J., M. Ledwidge, J. Gallagher, C. Keenan and C. Ryan (2022). "Pharmacogenetic interventions to improve outcomes in patients with multimorbidity or prescribed polypharmacy: a systematic review." Pharmacogenomics Journal **22**(2): 89-99.

Okusanya, B., L. J. Kimaru, N. Mantina, L. B. Gerald, S. Pettygrove, D. Taren and J. Ehiri (2022). "Interventions to increase early infant diagnosis of HIV infection: A systematic review and meta-analysis." PLoS ONE **17**(2): e0258863.

Osborn, R. L., A. C. Demoncada and M. Feuerstein (2006). "Psychosocial interventions for depression, anxiety, and quality of life in cancer survivors: meta-analyses." International journal of psychiatry in medicine **36**(1): 13-34.

Ouwens, M., M. Hulscher, R. Hermens, M. Faber, H. Marres, H. Wollersheim and R. Grol (2009). "Implementation of integrated care for patients with cancer: a systematic review of interventions and effects." Int J Qual Health Care **21**(2): 137-144.

Panagioti, M., P. Bower, E. Kontopantelis, K. Lovell, S. Gilbody, W. Waheed, C. Dickens, J. Archer, G. Simon, K. Ell, J. C. Huffman, D. A. Richards, C. van der Feltz-Cornelis, D. A. Adler, M. Bruce, M. Buszewicz, M. G. Cole, K. W. Davidson, P. de Jonge, J. Gensichen, K. Huijbregts, M. Menchetti, V. Patel, B. Rollman, J. Shaffer, M. C. Zijlstra-Vlasveld and P. A. Coventry (2016). "Association Between Chronic Physical Conditions and the Effectiveness of Collaborative Care for Depression: An Individual Participant Data Meta-analysis." JAMA psychiatry **73**(9): 978-989.

Patel, A. and M. Jafferany (2020). "Multidisciplinary and Holistic Models of Care for Patients With Dermatologic Disease and Psychosocial Comorbidity: A Systematic Review." JAMA Dermatol **156**(6): 686-694.

Patel, P., C. E. Rose, P. Y. Collins, B. Nuche-Berenguer, V. V. Sahasrabuddhe, E. Peprah, S. Vorkoper, S. D. Pastakia, D. Rausch, N. S. Levitt and N. H. N. P. D. C. T. O. Group (2018). "Noncommunicable diseases among HIV-infected persons in low-income and middle-income countries: a systematic review and meta-analysis." AIDS (London, England) **32 Suppl 1**: S5-S20.

Poot, C. C., E. Meijer, A. L. Kruis, N. Smidt, N. H. Chavannes and P. J. Honkoop (2021). "Integrated disease management interventions for patients with chronic obstructive pulmonary disease." Cochrane Database Syst Rev **9**(9): Cd009437.

Robb, S. L. and D. Hanson-Abromeit (2014). "A review of supportive care interventions to manage distress in young children with cancer and parents." Cancer Nursing **37**(4): E1-E26.

Robertson, J., S. Baines, E. Emerson and C. Hatton (2017). "Service Responses to People with Intellectual Disabilities and Epilepsy: A Systematic Review." Journal of applied research in intellectual disabilities : JARID **30**(1): 1-32.

Rutledge, T., L. S. Redwine, S. E. Linke and P. J. Mills (2013). "A meta-analysis of mental health treatments and cardiac rehabilitation for improving clinical outcomes and depression among patients with coronary heart disease." Psychosomatic medicine **75**(4): 335-349.

Sabri, B. and A. Gielen (2019). "Integrated Multicomponent Interventions for Safety and Health Risks Among Black Female Survivors of Violence: A Systematic Review." Trauma Violence Abuse **20**(5): 720-731.

Savage, E., J. Hegarty, E. Weathers, L. Mulligan, C. Bradley, C. Condon, J. Cronly, E. Lehane, A. O. Reilly, I. Hartigan, A. Horgan, J. Browne, M. Vera, M. Aileen, C. Jodi, F. Moira and D. Jonathan (2016). "Transforming Chronic Illness Management through Integrated Care: A Systematic Review of What Works Best and Why." International Journal of Integrated Care (IJIC) **16**(6): 1-2.

Shaw, J., S. Sethi, L. Vaccaro, L. Beatty, L. Kirsten, D. Kissane, B. Kelly, G. Mitchell, K. Sherman and J. Turner (2019). "Is care really shared? A systematic review of collaborative care (shared care) interventions for adult cancer patients with depression." BMC health services research **19**(1): 120.

Smith, S. M., H. Soubhi, M. Fortin, C. Hudon and T. O'Dowd (2012). "Managing patients with multimorbidity: Systematic review of interventions in primary care and community settings." BMJ (Online) **345**(7874): e5205.

Socías, M. E., M. Karamouzian, S. Parent, J. Barletta, K. Bird and L. Ti (2019). "Integrated models of care for people who inject drugs and live with hepatitis C virus: A systematic review." Int J Drug Policy **72**: 146-159.

Thombs, B. D., P. de Jonge, J. C. Coyne, M. A. Whooley, N. Frasure-Smith, A. J. Mitchell, M. Zuidersma, C. Eze-Nliam, B. B. Lima, C. G. Smith, K. Soderlund and R. C. Ziegelstein (2008). "Depression screening and patient outcomes in cardiovascular care: a systematic review." Jama **300**(18): 2161-2171.

Uyei, J., D. Coetzee, J. Macinko and S. Guttmacher (2011). Integrated delivery of HIV and tuberculosis services in Sub-Saharan Africa: A systematic review.

Vermunt, N., M. Harmsen, G. P. Westert, M. G. M. Olde Rikkert and M. J. Faber (2017). "Collaborative goal setting with elderly patients with chronic disease or multimorbidity: a systematic review." BMC geriatrics **17**(1): 167.

Zhao, Y., Y. Ma, C. Zhao, J. Lu, H. Jiang, Y. Cao and Y. Xu (2022). "The effect of integrated health care in patients with hypertension and diabetes: a systematic review and meta-analysis." BMC Health Serv Res **22**(1): 603.
