# Supplementary material for: Models of integrated care for multi-morbidity assessed in systematic reviews: a scoping review
Source: BMC Health Serv Res. 2023 Aug 23;23:894. doi: 10.1186/s12913-023-09894-7 (PMC10463690; doi:10.1186/s12913-023-09894-7)
Supplement: Supplementary file 3 — Supplementary Material 3 [file 12913_2023_9894_MOESM3_ESM.pdf]

Additional file 3: Definitions of integrated care as reported in included reviews

Study ID	Definition
Atlantis 2014 (20)	"Coordinated care with multidisciplinary model of care."
Bulstra 2021 (33)	"In the broadest sense, integration is the joining of 2 or more health services that were previously separated in some way (for instance, delivered by different health workers or at different locations). The specific integration that was the topic of our systematic review and meta-analysis is the joining of health services for HIV and at least one other disease or condition."
Chuah 2017 (21)	"Managerial or operational changes to health systems to bring together inputs, delivery, management and organization of particular service functions as a means of improving coverage, access, quality, acceptability and (cost)-effectiveness. This may include:<br>• Service integration: interventions that combine 'different packages of services'<br>• Integration of service delivery points which include health units of any type for e.g. primary care settings, hospitals, residential settings, service organizations etc.<br>• Integration at different levels of service delivery: macro-, meso-, micro-levels<br>• Process modifications to facilitate integration for e.g. referral and linkage mechanisms or standard operating procedures<br>• Introduction of technologies aimed at aiding integration<br>• Integration of management decisions".
Dudley 2011 (22)	"Any management or organisational change strategy applied to existing systems that aimed to increase integration at the service delivery level in primary health."
Haldane 2018 (23)	"Managerial or operational changes to health systems to bring together inputs, delivery, management and organization of particular service functions as a means of improving coverage, access, quality, acceptability and (cost)-effectiveness. This may include: Service integrations that combine "different packages of services'' such as integration of service delivery points, integration at different levels of service delivery, process modifications, introduction of technologies aimed at aiding integration, integration of management decisions."
Hopman 2016 (24)	Comprehensive care: "those initiatives that proactively seek to structure and coordinate care and improve health outcomes while constraining health care expenditures. Many different terms are being applied to comprehensive care including integrated care, guided care, case management, and shared care. Core elements of these initiatives are: (a) a well-coordinated and proactive approach to health and social care needs, often including comprehensive needs assessment, (b) patient-centeredness by involving patients in decision-making and planning their care process, and by taking their individual needs into account, (c) (simultaneous) delivery of multiple interventions, and (d) involvement of professionals from multiple disciplines." Integrated care defined as "those initiatives that proactively seek to structure and coordinate care and improve health outcomes while constraining health care expenditures."
Huang 2013 (25)	"Collaborative care points out coordinated care management in primary practices, which involves primary care physicians, nurses and other specialists or professionals who provided patient-orientated and guideline-based management to patients at the primary care level."
John 2020 (34)	"Patient-Centered medical home (PCMH) model is one of the chronic care models (CCM). It typically consists of a general practitioner (GP)-led care, as part of a multidisciplinary team (MDT) that aims to provide patient-centred care that is also comprehensive and coordinated, with emphasis on self-management and patient education."

Kadia 2021(35)	"Various approaches of delivering integrated services have been proposed and vary from having the services within one health facility to a one-stop-shop strategy in which the services are provided as a single package by the same healthcare team."
Kappelin 2021(36)	"Multiprofessional approach to patient care involving a Care Manager (CM), who can be a nurse, psychologist, social worker, or other health care professional, collaborating with another medical health professional, often a PC physician; the CM follows a structured management plan including medication and/or psychological treatment; the CM has scheduled patient follow-ups with the patient face-to-face, by telephone or digitally; and enhanced interprofessional communication between the CM and the PC physician."
Kastner 2018 (26)	Definition not reported. Multi-morbidity interventions: "those that integrate the care of 2 or more high-burden chronic diseases".
Lee 2021 (37)	Interprofessional collaborative practice (ICP) as a situation in which "multiple health workers from different professional backgrounds work together with patients, families, carers, and communities to deliver the highest quality of care."
Li 2017 (27)	"Multicomponent strategies characterized by active collaboration between specialist and primary care providers, assisted by a care manager, and typically including measurement-based care."
Martens 2021 (38)	"Specific modifications to healthcare organization, models of collaborative care and care coordination for persons with a severe mental illness (SMI) in a community mental health setting (CMHS)."
Nyirenda 2022 (39)	"A variety of managerial or operational changes to health systems to bring together inputs, delivery, management and organisation of particular service functions" In integrated care, a patient receives a complete care for two or more health problems or diseases at one service point."
Rohwer 2021 (40)	"Full integration of service delivery was defined as models where patients (primarily treated for diabetes, hypertension or any other disease) received the full package of care (prevention, diagnosis and treatment) for diabetes or hypertension and any other chronic disease at the same point of care by one or more healthcare professionals. Partial integration of services was defined as models where patients treated for diabetes, hypertension or any other chronic disease received part of the package of care (either prevention, diagnosis or treatment) for another disease."
Sigfrid 2017 (28)	Domains of integration:<br>● Integration across disease programs (clinically related diseases)<br>● Integration between vertical (disease-specific) and horizontal (system-wide) programs<br>● Integration across public health programs and health service interventions<br>● Integration across activities in the health systems and other sectors
Smith 2021a (29)	Integrated care not defined per se. Primary Health care was defined as providing: "integrated, easy to access, health care services by clinicians who are accountable for addressing a large majority of personal health care needs, developing a sustained and continuous relationship with patients, and practicing in the context of family and community"
Smith 2021b (41)	"Any type of intervention based in primary care and community settings that was specifically directed towards a group of people defined as having multimorbidity." Primary healthcare was defined as providing "integrated, easy to access, healthcare services by clinicians who are accountable for addressing a large majority of personal healthcare needs, developing a sustained and continuous relationship with patients, and practising in the context of family and community."

Tully 2015 (30)	“Collaborative care intervention, defined as a coordinated model of care involving multidisciplinary health-care providers, including:<br>● at least one health professional (eg, nurse, psychiatrist, psychologist) in addition to the primary care physician;<br>● a structured patient management plan that delivers either a pharmacological or a non-pharmacological depression intervention;<br>● scheduled patient follow-up and<br>● enhanced inter-professional communication between the multi-professional team.<br>This may include usual coronary heart disease care or blended depression-coronary heart disease care.”
Van Eck van der Sluijs 2018 (31)	Collaborative care: "a treatment intervention performed by a multidisciplinary team with at least two of the following three care givers involved: a medical doctor (GP or medical specialist), consultant psychiatrist and/or a case manager who is most often a nurse.”
Watson 2013 (32)	“Primary care providers and mental health providers working together to address the comprehensive needs of the patient.”
