# Supplementary material for: Models of integrated care for multi-morbidity assessed in systematic reviews: a scoping review
Source: BMC Health Serv Res. 2023 Aug 23;23:894. doi: 10.1186/s12913-023-09894-7 (PMC10463690; doi:10.1186/s12913-023-09894-7)
Supplement: Supplementary file 4 — Supplementary Material 4 [file 12913_2023_9894_MOESM4_ESM.pdf]

Additional file 4: Components of integrated care (detailed table)

Study ID	Conditions	Components of integrated care related to:				
		Where services were provided	Types of services provided	Health professionals involved in care	Coordination and organisation of care	Involvement of patients in care
Systematic reviews with included studies from LMICs						
Bulstra 2021 (34)	HIV and TB, NCDs, mental health, primary health care	Single facility:<br>• One-stop-shop<br>• Co-location<br><br>Multiple facilities	HIV:<br>• Prevention services<br>• Screening<br>• HIV testing<br>• CD4 and viral load testing<br>• HIV self-testing<br>• Home-based HIV testing and counselling<br>• Linkage to ART care<br>• ART<br>• ART adherence counselling<br>• Pre-exposure prophylaxis<br><br>NCDs:<br>• NCD screening<br>• Hypertension screening<br>• CVD screening<br>• Psychological support<br><br>Mental health:<br>• Depression care<br>• Depression screening<br>• Mental health care<br><br>TB:	Not reported	Not reported	Not reported

			• TB testing<br>• TB Intensive case finding<br>• TB treatment<br><br>Cancer:<br>• Colorectal and breast cancer screening<br>• Cervical cancer screening<br><br>General:<br>• Primary health care<br>• Chronic pain relief therapy<br>• Chronic disease management			
Dudley 2011(23)	HIV and TB	Single facility (TB clinics)	HIV prevention and control<br><br>HIV testing and counselling<br><br>TB treatment	Health workers	Not reported	Not reported
Kadia 2021 (36)	HIV and TB	Single facility	Treatment for HIV and TB	Not reported	Not reported	Not reported
Nyirenda 2022 (40)	Tuberculosis and Diabetes	Single facility	Screening of TB in patients with Diabetes<br><br>Screening of Diabetes in patients with TB<br><br>Bi-directional screening	Not reported	Not reported	Not reported
Rohwer 2021 (41)	Diabetes and/or hypertension and other	Single facility:<br>• Co-location<br>• One-stop-shop	Diabetes:<br>• Testing<br>• Treatment<br><br>Hypertension:	Primary health care nurses<br><br>Physicians	Training of healthcare providers<br><br>Appointments to initiate ART	Appointment reminders (letters, phone, sms)<br><br>Support hotline

	diseases (HIV, depression)	Community	• Testing<br>• Treatment<br><br>HIV:<br>• Testing<br>• Linkage to care<br>• ART<br><br>Depression:<br>• Screening<br>• Management<br><br>General:<br>• Health promotion and education	Ward-based outreach teams	Designated chronic care area at facility<br><br>Supply of critical medicines<br><br>Pre-packaging of medications<br><br>Clinical management support (treatment guidelines, algorithms, electronic decision-support)<br><br>Expanded prescribing provisions for nurses<br><br>Key treatment messages displayed<br><br>Onsite supervision/support<br><br>Additional staff<br><br>Appropriate referral	Educational pamphlets
Sigfrid 2017(29)	HIV and cervical cancer	Single facility:<br>• Within clinic (one-stop shop)	Cervical cancer screening<br><br>Treatment of minor lesions<br><br>Treatment of larger lesions<br><br>Referral<br><br>Education and counselling<br><br>Outreach awareness campaign	not reported	Coordination of local experts and pathologists	not reported

		Single facility:<br>• Co-location (clinics at same facility)	Counselled and referred for screening<br><br>Cervical cancer screening<br><br>Treatment of minor lesions<br><br>Referral of any treatment<br><br>Referral of complex lesions	HIV and gynecological specialists	Coordination of ART and reproductive units with bidirectional referral and patient tracking<br><br>Coordination of mobile unit and ART clinic<br><br>Coordination between HIV and Cancer screening clinics<br><br>Care coordinated by specialist nurse	not reported
		Multiple facilities	Cervical cancer screening<br><br>Treatment of minor lesions<br><br>Treatment of larger lesions<br><br>Referral<br><br>Counselling<br><br>HIV screening and referral<br><br>Community awareness for Cervical cancer screening	not reported	Coordination with external center and lay health workers to ensure follow-up<br><br>Coordinated referral between reproductive and child health and HIV clinics<br><br>Coordination with external pathologists<br><br>Coordinated referral and follow-up	not reported
Systematic reviews with included studies from low-, middle-, and high-income countries						
Chuah 2017(22)	HIV and mental health	Single facility	Not reported	Not reported	Case conferences<br><br>Shared medical notes and individual discussions<br><br>Internal referral system	Not reported

					Joint consultations	
		Multiple facilities	Referral between services	Not reported	Collaborative networks and referral mechanisms<br><br>Case discussions	Not reported
		Not reported	For mental health patients:<br><br>Referral to HIV services	Nurse<br><br>Primary care staff<br><br>Social worker<br><br>Depression-care manager	Case management	Patient-led coordination of care
Haldane 2018(24)	HIV and cardiovascular disease, hypertension or diabetes	Single facility	Treatment<br><br>Screening and referral	multi-disciplinary teams of doctors, nurses, community workers, nutritionists, AIDS officers and pharmacists<br><br>community health workers	medical record tagging	Patient perspectives<br><br>Self-management
John 2020 (35)	Various conditions including Diabetes, Hypertension, depression, coronary heart disease	Not reported	Not reported	Multidisciplinary team	Coordinated care<br><br>Continuity of care<br><br>Data driven quality of care	Patient engagement<br><br>Self-management plans
Lee 2021 (38)	Diabetes and hypertension	Single facility:<br>• Co-location	Group educational sessions	MDT including: Physician, endocrinologist, dietician, nutritionist, pharmacists, physical	Regular team meetings (bi-weekly, weekly, daily)<br><br>Shared medical appointments	Communication (face-to-face, online, phone calls)

				therapist, psychologist, nurse, diabetes specialist, educator, exercise specialist, social worker, pharmacists, optometry faculty and students,	Shared electronic medical records<br><br>Case management<br><br>Collaborative practice agreement<br><br>Treatment plans<br><br>Consultant services<br><br>Caseload tracking<br><br>Clinical practice guidelines<br><br>Medication reconciliation	Patient-centred medical home
Systematic reviews with included studies from high-income countries						
Atlantis 2014(21)	Diabetes and depression	Not reported	Treatment maintenance and relapse prevention<br><br>Referral<br><br>Education	Nurse<br><br>Psychologist<br><br>Nurse plus general practitioner	Stepped care framework<br><br>Supervised case-manager	Problem-solving therapy<br><br>Goal setting<br><br>Self-monitoring education<br><br>Self-care education<br><br>Patient-centered care
Hopman 2016(25)	Multiple chronic conditions	Home<br><br>Single facility	Not reported	Medical groups and physician networks<br><br>Nurse<br><br>General practitioner	Case manager<br><br>Follow up appointments<br><br>Prevention of hospital readmission<br><br>Decision support<br><br>Clinical information system<br><br>Delivery system design	Self-management<br><br>Community resources

Huang 2013(26)	Depression and diabetes	Single facility	Treatment for depression<br><br>Management of diabetes	MDT: primary care physician, nurse, psychologist	Structured management plan<br><br>Enhanced Interprofessional communication<br><br>scheduled follow-up	Not reported
Kappelin 2021 (37)	Diabetes, COPD, Congestive heart disease, Asthma, Musculoskeletal pain, Acute coronary heart syndrome,	Primary care or community setting	Psychological treatment	Physician or advanced practice nurse, plus a care manager (social work specialist, research coordinator, psychologist, problem-solving technique specialist, nurse)	Structured management plan (stepped care model, care plan, education)<br><br>Scheduled follow-up appointments (telephonic or face-to-face)<br><br>Enhanced interprofessional communication (written or oral)	Support for self-care<br><br>Problem-solving techniques
Kastner 2018(27)	Diabetes and depression, diabetes and cardiovascular disease, heart failure and COPD	PHC facilities<br><br>Home<br><br>Outpatient clinics<br><br>Hospitals<br><br>Nursing homes	Education<br><br>Cognitive behavioural therapy	Multi-disciplinary teams	Case management<br><br>Disease management<br><br>Care pathway<br><br>Facilitated relay<br><br>Electronic clinical decision support system<br><br>Computer-based counselling system<br><br>Telemedicine<br><br>Telecare	Self-management<br><br>Patient empowerment<br><br>Medication self-management
Li 2017(28)	Cancer and depression	Home	Therapy for depression	not reported	Telephone monitoring and relapse prevention	Problem-solving therapy

					Automatic symptom monitoring<br><br>Follow-up	
Martens 2021 (39)	Severe mental illness and various other chronic diseases (Diabetes, CVD risk factors, dyslipidemia)	Not reported	Screening<br>Counselling<br>Psychological treatment<br>Behavioural interventions	MDT<br>Nurse care manager<br>Psychiatrist,<br>Advanced practice nurse<br>Endocrinologist consultant<br>Peer specialists	Team meetings<br>Training of team<br>Individualised health plan<br>Coordination of care<br>Case manager<br>Regular team meetings<br>Care management	Education materials<br>Health education<br>Support to attend medical appointments<br>Self-management support<br>Lifestyle coaching<br>Shared care-plans<br>Peer coaching<br>Peer support
Smith 2021a(30)	Multiple chronic conditions	Home-based care<br><br>Multiple facilities<br><br>Single facility	Diagnosis<br><br>Treatment<br><br>Education<br><br>Preventive care<br><br>Pharmaceutical care<br><br>Cognitive behavioural therapy	Family physician<br><br>Nurse<br><br>Occupational therapist<br><br>Physical therapist<br><br>Primary care practitioner<br><br>Psychologist<br><br>Dietician<br><br>Social worker<br><br>Endocrinologist<br><br>Psychiatrist	Case management<br><br>Regular planned<br><br>participant visits<br><br>Weekly team meetings<br><br>Stepped care<br><br>Peer support through group meetings<br><br>Multidisciplinary team management<br><br>Structured visits with nurses<br><br>Individualised care plans and treatment targets,	Goal setting<br><br>Self-management support<br><br>Individualised care plans<br><br>Patient engagement workshop<br><br>Patient-oriented intervention

					Follow up phone calls	
Smith 2021b (42)	Multimorbidity (various conditions)	Not reported	Not reported	MDT<br>Nurses<br>Physicians<br>Chronic disease and management professionals (Nursing, physical activity, nutrition, respiratory therapy, smoking cessation)<br>Coaches	Home assessments<br>Coordination of care<br>Monthly monitoring Patient care plans<br>Group practices<br>Structured extended GP consultations and relationship continuity<br>Practitioner support and training<br>Continuity of care<br>IT support<br>GP training<br>Medication review	Self-management support<br>Shared care plan<br>Goal setting<br>Focus on patient priorities<br>Education materials<br>Telephone coaching<br>Workshop and telephone calls before and after medical appointment
Tully 2015(31)	Coronary heart disease and Depression	Not reported	Treatment for depression: Problem-solving therapy, Cognitive behavioural therapy, Pharmacotherapy<br>Education<br>Referral to community mental health service	Allied health team<br>Nurse<br>Social worker<br>Primary care physician<br>Psychiatrist<br>Physician	Telephone follow-up<br>Stepped care approach<br>Structured follow-up<br>Feedback to PCP	Patient preferences for care<br>Development of patient goals
Van Eck van der Sluijs 2018(32)	Chronic diseases and depression or dysthymia	Single facility	Diagnosis<br>Treatment for chronic condition	Case manager<br>General practitioner<br>Psychiatrist	Case management	Not reported

			Monitoring or psychotherapy for depression/anxiety			
Watson 2013(33)	Chronic diseases and depression	Not reported	Treatment for depression: Pharmacotherapy, Problem-solving therapy, Cognitive behavioural therapy<br><br>Education<br><br>Referral to mental<br><br>services	Nurse<br><br>Psychologist<br><br>Psychiatrist supervision<br><br>Social worker<br><br>MDT	Stepped care approach<br><br>Monitoring of treatment response	Patient preference for treatment<br><br>Decision support<br><br>Goal setting<br><br>Support for self-care<br><br>Motivational coaching
