# Supplementary material for: Models of integrated care for multi-morbidity assessed in systematic reviews: a scoping review
Source: BMC Health Serv Res. 2023 Aug 23;23:894. doi: 10.1186/s12913-023-09894-7 (PMC10463690; doi:10.1186/s12913-023-09894-7)
Supplement: Supplementary file 5 — Supplementary Material 5 [file 12913_2023_9894_MOESM5_ESM.pdf]

Additional file 5: Verbatim conclusion on process and health outcomes of included studies

Study ID	Reported findings on process outcomes	Reported findings on health outcomes
**Reviews with included studies from LMICs**		
Bulstra 2021 (34)	"The evidence indicates that integration of HIV testing and counselling services into non-HIV health programmes for people at risk of acquiring HIV, and integration of non-HIV services into ART programmes for PLHIV, tends to lead to improved service uptake and health benefits for HIV and other diseases or conditions. However, the effects of integrating HIV services into broader health systems and the economic impacts of integration are less clear and require further study."	"The evidence indicates that integration of HIV testing and counselling services into non-HIV health programmes for people at risk of acquiring HIV, and integration of non-HIV services into ART programmes for PLHIV, tends to lead to improved service uptake and health benefits for HIV and other diseases or conditions."
Dudley 2011 (23)	"Adding on provider initiated counselling and testing to tuberculosis and sexually transmitted infection primary health care clinic services probably increases the number of people receiving HIV counselling and HIV testing."	Not reported
Kadia 2021 (36)	Not reported	"The pooled ART uptake in this study was suboptimal even when compared to ART uptake in general HIV population. This suggests that programmes integrating treatment of TB and HIV in SSA do not, in general, achieve high uptake of ART among co-infected patients. Nonetheless, recent studies tended to show much higher uptake than older studies and this may indicate an overall improvement in ART uptake in recent times."
Nyirenda 2022 (40)	Not reported	"The synthesized evidence is weak to support the effect of TB and DM integrated care on screening coverage and treatment loss to follow-up. Nevertheless, the findings still suggested that TB/DM integrated care could have an impact on bidirectional screening and treatment loss to follow-up in both TB patients and people with DM."
Rohwer 2021 (41)	Not reported	"The evidence on the effectiveness of integrated models of care for people with diabetes, hypertension and other comorbidities, on health outcomes is very uncertain. We therefore do not know whether integrated models of

		care lead to better or worse outcomes, or may make no difference at all among people with diabetes, hypertension and other chronic conditions.”
Sigfrid 2017 (29)	“Evidence suggests that the provision of integrated services was feasible, safe, and acceptable to both staff and women attending the health facilities. Uptake of cervical cancer and HIV screening was high in all models described, but loss to follow-up for cervical cancer treatment was a challenge in most studies.”	Not reported
**Reviews with included studies from LMICs and HICs**		
Chuah 2017 (22)	“Single-site integration augments multidisciplinary coordination while reducing access barriers, but can be difficult to implement when a fuller continuum of specialized care involving multiple treatment modalities is needed particularly in low-resource settings.”<br>“Multi-facility integration may comprehensively serve multi-morbid patients, but appropriate coordination and referral mechanisms are crucial to prevent fragmented care.”<br>“Active case management by non-clinicians offers considerable potential especially in low resource settings with shortages of mental health specialists although appropriate training and support is essential.”<br>“Involving the patients not just as service users but also as active partners in improving integration within the treatment process,is a promising approach”	Not reported
Haldane 2018 (24)	“Studies showed that CVD and HIV service integration is feasible.”	Not reported
John 2020 (35)	Not reported	“Current evidence suggests that PCMH-based care showed significant improvements in depression, HRQoL, self-management, biomedical, and health utilisation outcomes compared to standard GP care.”

Lee 2021 (38)	Not reported	"The results of this systematic review and meta-analysis suggest that there is a positive association of ICP in primary care with HbA1c, SBP, and DBP levels in adult patients with diabetes or hypertension."
**Reviews with included studies from HICs**		
Atlantis 2014 (21)	Not reported	"Collaborative care significantly improved the depression score and HbA1c level compared with control conditions. Depression remission did not predict better glycaemic control across studies."
Hopman 2016 (25)	"There is no evidence that comprehensive care reduces the number of primary care or GP visits or healthcare costs."	"Providing comprehensive care might result in more patient satisfaction, less depressive symptoms, a better health-related quality of life or functioning of multimorbid or frail patients, but the evidence is insufficient."
Huang 2013 (26)	Not reported	"Collaborative care model significantly improves depression outcomes and adherence to medication in diabetic patients with depression, comparing with usual care."
Kappelin 2021 (37)	Not reported	"The results from 11 studies show that CC effectively decreases depressive symptoms in patients with multimorbidity involving depression and 1 somatic disease in studies with medium-to-high quality. No conclusions can be drawn regarding the effectiveness of CC in patients with multimorbidity involving anxiety, or for patients with multimorbidity involving depression and 2 or more somatic diseases."
Kastner 2018 (27)	"The intervention combination of care pathways and education significantly increased use of mental health services in those with [DM + (depression or CVD)]."	"The intervention combination of case management + education + self-management significantly reduced depressive symptoms in older adults with [depression + COPD] or [DM + CVD], and reduced HbA1c levels in those with [DM + another disease]."<br>"Care-coordination or telemedicine interventions that included at least education as a component significantly reduced dyspnea-related disability and improved cognitive functioning in patients with [DM + depression] or [COPD + HF]."

Li 2017 (28)	Not reported	"Collaborative care resulted in significantly better standardized mean depression scores compared with usual care, and this was sustained up to 12 months after initiation of the intervention."<br><br>"Cancer patients with depression may benefit from pharmacological or psychological interventions, without evidence for the superiority of any specific treatment over another."
Martens 2021 (39)	Not reported	"The literature shows modest effects on quality of life, but not consistently over different studies. No clear outcomes were shown, although it seems a more integrated approach has a positive effect on health outcomes and Health related Quality of Care, and the use of peer-interventions could improve commitment to health services."
Smith 2021a (30)	"The intervention may make little or no difference to health service use (low certainty evidence), may slightly improve medication adherence (low certainty evidence), probably slightly improves patient related health behaviours (moderate certainty evidence), and probably improves provider behaviour in terms of prescribing behaviour and quality of care (moderate certainty evidence). Cost data were limited."	"There was little or no difference in clinical outcomes (based on moderate certainty evidence)."<br><br>"Mental health outcomes improved (based on high certainty evidence) and there were modest reductions in mean depression scores for the comorbidity studies that targeted participants with depression."<br><br>"There was probably a small improvement in patient-reported outcomes (moderate certainty evidence) although two studies that specifically targeted functional difficulties in participants had positive effects on functional outcomes with one of these studies also reporting a reduction in mortality at four year follow-up."
Smith 2021b (42)	Not reported	"Despite the number of randomized controlled trials, there are remaining uncertainties about the effectiveness of interventions for people with multimorbidity."
Tully 2015 (31)	Not reported	"Collaborative depression care in the CHD population did not lead to a sustained reduction in MACE. Small reductions in depressive symptoms were evident for CC and intervention participants were more likely to achieve depression remission. Small effect sizes for anxiety symptom reduction and improvement in mental QOL were evident with CC"<br><br>"The findings did not suggest a significant benefit for physical QOL or

		healthcare costs.”
Van Eck van der Sluijs 2018 (32)	Not reported	“CC is more effective than CAU in terms of illness burden and physical outcomes for patients with comorbid depressive disorder and chronic medical conditions, particularly in patients with hypertension, HIV, COPD, multi-morbidity, arthritis, cancer and ACS.”<br>“CC is associated with more diagnostic and treatment intervention procedures, and hence, may play a role in improving quality of life.”<br>“Compared to CAU, CC is also effective for depression outcomes in patients with chronic medical conditions.”
Watson 2013 (33)	Not reported	“Collaborative care interventions improved outcomes for depression and quality of life in primary care patients with varying medical conditions”
