# Supplementary material for: Models of integrated care for multi-morbidity assessed in systematic reviews: a scoping review
Source: BMC Health Serv Res. 2023 Aug 23;23:894. doi: 10.1186/s12913-023-09894-7 (PMC10463690; doi:10.1186/s12913-023-09894-7)
Supplement: Supplementary file 7 — Supplementary Material 7 [file 12913_2023_9894_MOESM7_ESM.docx]

**Additional files:**

Additional file 1: Search strategies for electronic databases

Additional file 2: Excluded studies

Additional file 3: Definitions of integrated care as reported in included reviews

Additional file 4: Components of integrated care (detailed table)

Additional file 5: Verbatim conclusion on process and health outcomes of included studies

Additional file 6: PRISMA ScR Checklist
